# Durability Performance Investigation for Engineering Fiber Cementitious Composites (ECC): Review

**DOI:** 10.3390/polym15040931

**Published:** 2023-02-13

**Authors:** Ziyi Zhang, Yongcheng Ji, Wenhao Ji

**Affiliations:** 1 School of Aulin, Northeast Forestry University, Harbin 150040, China; 2 School of Civil Engineering, Northeast Forestry University, Harbin 150040, China

**Keywords:** engineering cement-based composites, content of fiber, water-binder ratio, durability, constitutive model

## Abstract

Engineered Cementitious Composite (ECC) is currently receiving more and more attention due to its excellent tensile strain hardening and multiple cracking properties. However, due to the high material cost of polyvinyl alcohol (PVA) fiber and quartz sand, its widespread promotion and application in the market are limited. Therefore, scholars at home and abroad have conducted many active studies on improving ECC. This paper summarizes the development history and research status of ECC materials, summarizes the current domestic and foreign researchers’ improvement methods for ECC materials, and classifies the improvement methods into three categories: the type of fiber variation, the water-binder ratio variation and adding mineral admixtures, the influences of the above three factors on the mechanical properties and durability of ECC, such as compressive and flexural resistance, are described in detail, and the mechanism of action is explained. Furthermore, this paper introduces the most common uniaxial compression and uniaxial tension constitutive models of ECC. They are briefly classified and evaluated, hoping to help readers’ numerical simulation analysis. Finally, some suggestions for ECC research, such as the proportion of water binders and the application of composite fibers, require further research.

## 1. Introduction

Concrete has gradually become an irreplaceable building material in civil engineering due to its abundant access to raw materials, strong plasticity in engineering, good working performance, ability to adapt to different uses and environments, durability, and other advantages [1]. With the development of the economy, cement concrete has been used in cities, highways, Bridges, tunnels, and other large-scale construction and has become an essential material for human social infrastructure construction. The first concrete material in the real sense of history can be traced back to ancient Roman times. In 70–80 AD, the ancient Romans began to build a concrete arena with fine processing and compaction of burnt lime, clay, gypsum, and lime plus volcanic ash [2]. Since then, the rapid development of concrete materials has experienced three stages: (1) the invention of Portland cement [3]. It is widely used in civil engineering with low cost, low energy consumption, accessible materials, and high strength advantages. (2) The use of steel reinforcement in concrete, the combination of steel reinforcement and concrete, is the decisive node in the history of concrete development. It solves the characteristics of ordinary concrete, such as solid compressive strength and weak tensile strength, influenced by its environment and poor structural durability. Parisian florist Monier [4] first put a chicken wire into a flowerpot made of cement mortar, creating a flowerpot with thin walls and high strength, known as the founder of a reinforced concrete structure. Since then, scholars worldwide have conducted scientific research and analysis on reinforced concrete. Coron [5] invented the calculation method of reinforced concrete and found that the two have a similar coefficient of thermal expansion and can be firmly combined without being affected by temperature. Frisina invented the theory of concrete shrinkage and creep, thus laying the foundation of prestressed steel structure concrete [6]. (3) The invention of prestressed concrete and the appearance of prestressed concrete enhanced the crack resistance and bearing capacity of concrete, further expanded its application scope, and laid the foundation for the emergence of modern high-rise buildings, such as the American Marina skyscraper [7] and Kresge Hall.

With the rapid development of urbanization and industrialization, the world population has grown exponentially [8]. The earth’s land resources are limited, and society has an increasing demand for the construction industry. People can only build long-span buildings, high-rise buildings, and super high-rise buildings to accommodate more population as much as possible [9]. Therefore, the realistic demand for the performance of concrete requires further improvement, not only to have good mechanical properties and durability but also to consider its seismic performance and frost resistance. Scholars at home and abroad have begun to conduct further research on concrete. There have been remarkable results. These include Ultra-high Performance Concrete (UHPC), Reactive Powder Concrete (Reactive Powder Concrete, RPC), and Fiber Reinforced Concrete (FRC) [10]. This type of concrete has excellent mechanical properties, durability, and permeability resistance, which can meet engineering applications in construction, road, bridge, and other fields. It has gradually been paid more attention to and applied in infrastructure projects.

In recent years, as the performance of FRC has shown strong excellence in the construction field, scholars at home and abroad began to modify and optimize them to obtain the characteristics of low cost and high strength to better and more widely benefit social undertakings. Currently, the modification of fiber-reinforced concrete can be divided into three categories: ① By changing the types of fiber mixed into engineering cementitious composites to change the characteristics. The current standard method is to mix polyvinyl alcohol fiber (PVA fiber), polypropylene fiber (PP fiber), carbon fiber (CF fiber), basalt fiber (BF fiber), and so on in concrete to improve the strength and crack resistance. ② The use of mineral admixtures instead of cement in engineering cementitious composites to improve performance. The current standard method is to use fly ash (FA), silica fume (SF), slag, and recycled powder (RP) to replace cement. ③ The fiber-reinforced concrete was modified with a coupling agent and other chemical substances.

Engineered Cementitious Composites (ECC) are one of the FRC materials proposed by Victor in 1992 [11]. Li used polyethylene (PE) fiber-reinforced cement mortar to prepare a cement-based composite material with high ductility [12]. ECC can also be called Strain-Hardening Cementitious Composites (SHCC) or Ultra High Toughness Cementitious Composite (UHTCC). Its components mainly include cement, fine aggregate, mineral admixture, fiber, and admixture, compared with conventional concrete to reduce the coarse aggregate. It has the advantages of crack resistance, impermeability, high deformation, and self-healing. The tensile strength of ECC can reach 4–6 MPa, and the ultimate tensile strain can reach 3–5%, which is several hundred times that of traditional concrete [13]. The appearance of ECC changes the characteristics of traditional cement-based material mortar and concrete, which are too broad and easy to break brittle.

However, despite its excellent mechanical properties and good durability, ECC removes coarse aggregate during preparation, increasing the amount of fine aggregate and cementitious material at a high cost. The most commonly used cemented material in traditional ECC is Portland cement. It will consume not only much limestone, sand, clay, and other natural resources but also have high energy consumption, significantly impacting the environment. The total emission of 5–7% [14] will aggravate the generation of the greenhouse effect, which is outside the current international background of green development and limits the sustainability of the large-scale use of ECC.

In order to reduce the cost of ECC and increase its green environmental performance, scholars have conducted much research on improving ECC. They change fiber types or add mineral admixtures to reduce manufacturing costs and increase their green environmental performance. Cui [15] replaced the toughening materials in traditional ECC with sisal fiber with green environmental protection and low price and prepared ECC materials that meet the requirements for the side walls of underground structures. Qiao et al. [16] used fly ash with different contents to replace the cementing material in ECC material to obtain ECC material with good performance. They found that when the fly ash content increased to 45% and 65%, the bending property of the material was greatly improved. Wang [17] et al. used Marine coral sand to replace quartz sand in ECC raw materials and obtained ECC materials with excellent mechanical properties, ductility, and crack control ability. Huang et al. [18] should prepare ECC sand with compressive strength over 130 MPa and tensile strain of up to 5%. Zhu et al. [19] used slag to improve ECC with high fly ash content. They found that the slag could effectively improve the compressive strength of ECC, especially the ECC with early compressive strength, and 30% granulated blast furnace slag (SL) and 40% fly ash (FA) had minimal drying shrinkage in the later period.

This paper has three novel points compared to the existing literature. Firstly, the admixtures of engineering fiber cementitious composites studied in this paper are expanded, which studies the properties of composites mixed with different fibers and mineral admixtures. This paper mainly analyzes two categories: using various mineral admixtures to replace cement in ECC; the recycled micro powder used to replace quartz sand in ECC. There are few studies on recycled micro powder and engineering fiber cementitious composite materials in the current open literature. Therefore, this paper further studies the composite materials mixed with recycled brick and concrete powder. Secondly, when analyzing the fiber types and content of engineering fiber cementitious composite materials, there are two characteristic analysis methods in this paper, for example, the transverse comparison between the compressive strength of the same fiber under different dosages and the compressive strength of different fiber under the exact dosage. Most of the existing literature research data focuses on one fiber, and this paper is innovative in providing a deeper understanding of engineering fiber cementitious composites. The third innovation point is the detailed study of the constitutive structure of ECC, such as the uniaxial compression constitutive model considering dimension and linear and nonlinear analysis models. The three innovative points will better show the performance and characteristics of engineering fiber cementitious composites so that readers will understand them more comprehensively.

This paper describes the research status of ECC materials, introduces the design theory of ECC materials in detail, summarizes the effects of fiber types, water-binder ratio, and mineral admixture types on the mechanical properties and durability of ECC materials, and elaborates on the mechanism of action in detail. Furthermore, the constitutive models of ECC uniaxial compression or tension are summarized and briefly evaluated. Finally, some suggestions on the future development direction of ECC are presented.

## 2. Compressive Strength

The compressive strength of cement base usually uses the cubic compressive strength of China GB 50010-2010 “Code for Design of Concrete structure” as the fundamental strength index of concrete, in the structural design is used as the prismatic axial compressive strength of concrete design index of compressive strength. Because the uniaxial compression test is relatively simple and accurate, most domestic and foreign scholars use the uniaxial compression test to study the compressive strength of ECC.

### 2.1. Type of Fiber

Fiber plays an essential role in high-performance concrete, and fiber incorporation can improve the mechanical properties of concrete by changing the cracking form [20]. Therefore, the influence of fiber types on the mechanical properties of ECC is also a hot research topic of scholars at home and abroad [21]. At the early stage of the research, Li [22] found that a new composite material made of PVA fiber (with a volume content of less than 2%) was added to cement concrete, which had high ductility and crack control ability. As early as the 1970s, Hughes et al. [23] studied the stress-strain curve of concrete reinforced with PP fiber mixed with fabricated and monofilament and pointed out that concrete toughened significantly after mixing PP fiber. Later, Ma et al. [24] studied the plastic cracking resistance and basic mechanical properties of modified polypropylene fiber cement-based composites and verified the conclusion of improving the flexural toughness of polypropylene fiber-reinforced concrete. Finally, Yuan et al. [25] proposed using PVA fiber to improve the problem of ECC cracks and found that adding PVA fiber to ECC could not only prevent the expansion of existing microcracks in the matrix but also effectively delay the emergence of new cracks. Later, Chao et al. [26] compared the strengthening effect of basalt fiber and polypropylene fiber on concrete. The results showed that, under the exact dosage, basalt fiber had better effects on the increase of compressive, flexural, and splitting tensile strength of concrete than polypropylene fiber. Jong et al. [27] compared BF, CF, and glass fiber (GF fiber) properties. The results showed that basalt had better alkali resistance and higher tensile strength than glass fiber, and basalt fiber had better thermal stability than carbon fiber and glass fiber. In summary, presently, domestic and foreign researchers mainly focus on PVA fiber, PP fiber, CF fiber, and BF fiber in studying fiber types in ECC performance.

The influence of fiber type on ECC performance mainly depends on the characteristics of the fiber itself. The polymerization of polyvinyl alcohol forms PVA fiber. Since the hydroxyl group of vinyl alcohol monomer is retained in the PVA molecule, the hydrophilicity of PVA fiber is good, which makes it have better dispersion than other fibers in the water-containing concrete mix [22]. Whether a new building structure or the repair and reinforcement of existing structures, the interface between fiber and concrete is its weakest part. The damage of the interface is preceded by the overall components, so good interface bonding performance is a prerequisite to ensure that the combined components and reinforced components work. The interface bonding performance determines the engineering structure’s safety, applicability, and durability [28]. Fibers can improve the toughness of cement and effectively improve the interface bonding performance. The mechanism of action is that fibers can reduce the shrinkage of the cement base. When the reinforced surface layer is thin, fibers can effectively reduce the risk of interface peeling and cracking caused by material shrinkage. When the composite material is subjected to a load, fibers can effectively inhibit the development of micro-cracks inside the cement base, reducing the stress around the primary crack redistribution, improving the residual stress after cracking, and changing the power transmission method and Forms of damage [29]. The bonding interaction between the hydroxyl group of polyvinyl alcohol and the cementitious group forming hydrogen bonds has excellent strength and stability. It is not affected by the matrix water-cement ratio and fiber type [30], which significantly increases the bonding strength between PVA fibers and cementite, resulting in good interfacial adhesion between PVA fibers and cementitious matrix.

**Figure 1:**
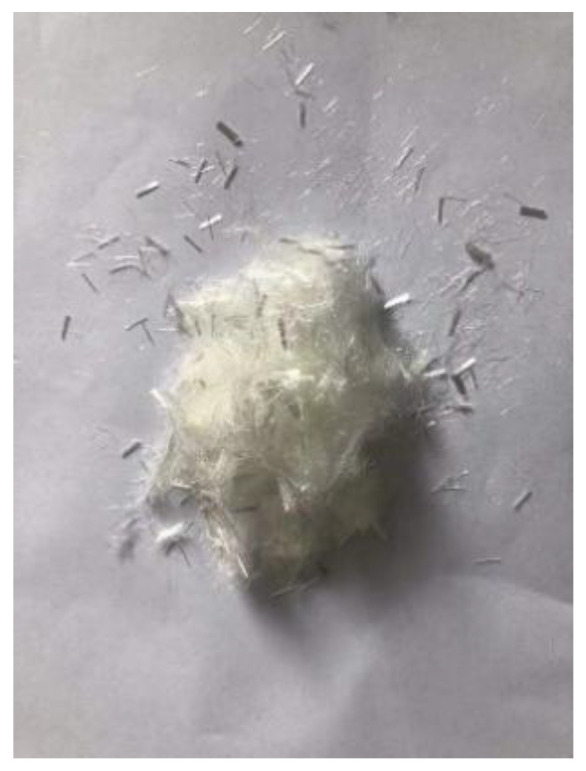
PVA fiber used in the review.

**Figure 2:**
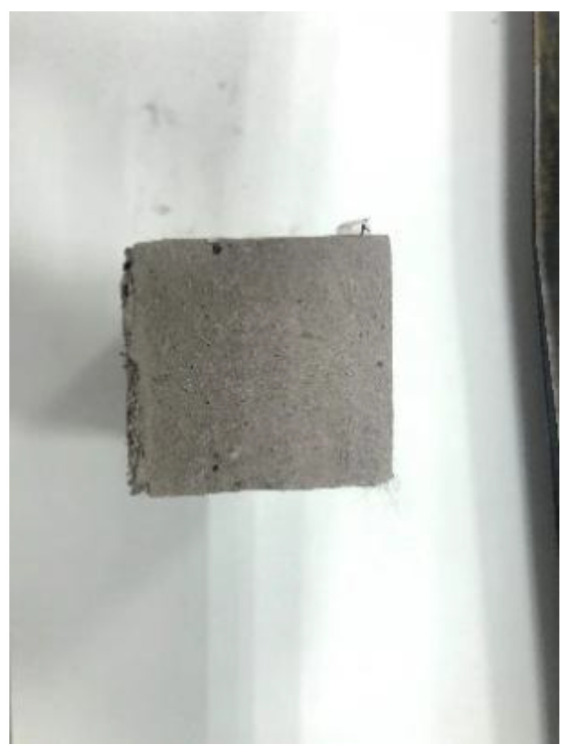
Finished PVA-ECC used in this review.

PP fiber is a low elastic modulus synthetic fiber with high strength, good ductility, excellent durability, and low price. Its raw material, the monomer C3H6, is a high molecular hydrocarbon, its chemical stability is good, and most chemicals do not react but has flammability, resulting in the reduction of the fire resistance of concrete. CF fiber is an organic fiber containing carbon. A graphite-like structure is formed in an inert atmosphere through multiple processes, such as pre-oxidation and high-temperature carbonization [31]. As a result, it has the advantages of high tensile strength and elastic modulus, stable chemical properties, and good bonding with concrete [32]. Basalt fiber is made from natural basalt ore at high temperatures. Compared with other standard fiber admixtures, it has higher tensile strength, deformation modulus, and corrosion resistance. In addition, it has the same mineral composition as basalt ores and can be degraded directly into the soil [33]. Figure 1 and Figure 2 show what these fibers used in the review look like before the fabricating process and for the composite. Moreover, the specific physical indicators are shown in Table 1.

#### 2.1.1. Compressive Strength of the Same Content of Fiber

**Figure 3:**
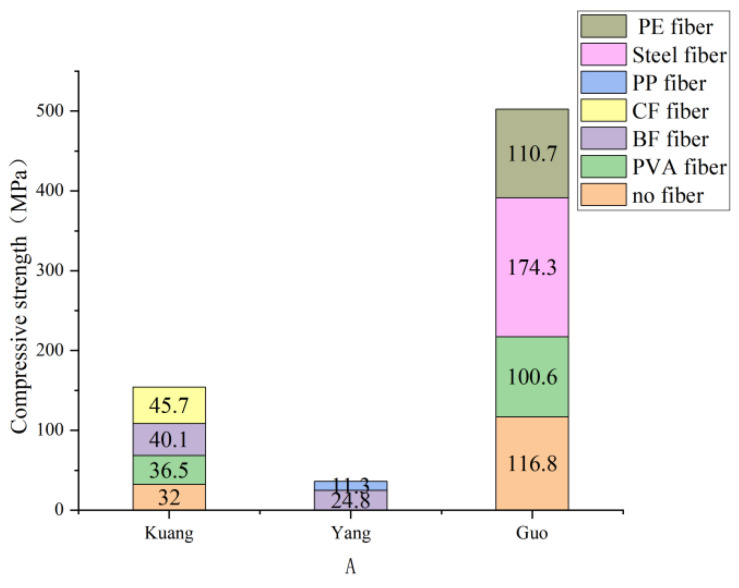
Compressive strength of the same mixing amount of fiber [41,42,43].

Kuang [41] made seven different fibers, including PVA-ECC, PP-ECC, CF-ECC, and BF-ECC, into specimens according to the fiber volume placement rate of 1.5% and conducted a cube compressive strength test. It is concluded that adding fiber can improve ECC’s compressive strength (mean value: 32.5–40 MPa) compared with the cement base without fiber (mean value: 32 MPa). Among them, PVA fiber (average compressive strength 36 MPa), basalt fiber (average compressive strength 37–39 MPa), and carbon fiber (average compressive strength 45 MPa) were significantly improved. Yang [42] conducted compressive experiments on polypropylene and basalt fiber and concluded that the compressive strength of the composite increased to a certain extent when the fiber content was lower than a specific range. However, the compressive strength of the composite gradually decreased after the fiber content increased to a specific range, mainly because the cement matrix’s effective compression zone decreased with the fiber content increase. In addition, the interface’s weak area increases with the fiber content. Guo [43], a foreign scholar, studied the compressive strength of three different types of fibers and conducted a uniaxial compressive test on specimens mixed with steel, PVA, and polyethylene fiber with the same volume fraction of 2%. It was found that specimens containing PE fiber have higher strain hardening ability than composites reinforced with PVA fiber or steel fiber. According to the Figure 3, composites containing PVA or steel fiber have higher strength than PE fiber-reinforced composites. In general, each fiber has a different critical volume rate. When the critical volume rate is not exceeded, the toughness of ECC will gradually increase with the increase of fiber content. The fiber clumping phenomenon will occur when the critical volume rate is exceeded.

#### 2.1.2. Compressive Strength of the Same Fiber Specimen with Different Dosage

**Figure 4:**
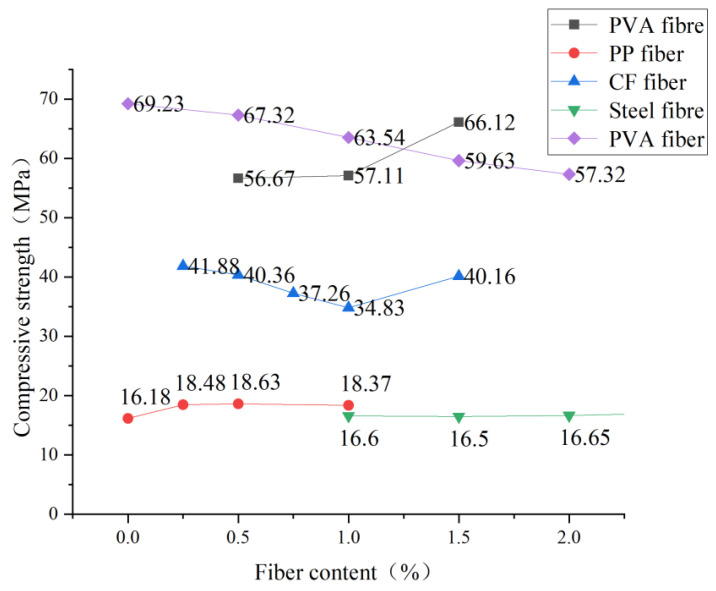
Compressive strength of the same fiber at different mixing amounts [44,45,46,47,48].

Scholars have made many achievements in the research on the compressive strength of different fibers mixed with ECC: Hao et al. [44] conducted compressive tests on ECC specimens with different contents of PVA. With the increase of the fiber content when fiber content increased from 0.5% to 1.0% and 1.5%, the 28-day compressive strength increased by 11.4% and 4.7%, respectively, and it was analyzed that the fibers were randomly distributed in cement-based composite materials. It is equivalent to adding many small reinforcing bars to the matrix. The bonding effect of fibers restrains the development of cracks in the matrix, significantly improves the ductility of the matrix, and improves the compressive strength of cement-based composites to a certain extent, which plays the role of fiber reinforcement. Xu [45] studied polypropylene fiber and found that the compressive strength of the comparison group mixed with polypropylene fiber was higher than that of the base group. However, when the mixing amount increased exponentially, the improvement could have been more apparent and tended to be gentle. Therefore, a high mixing amount could be achieved under a low fiber mixing. The content of polypropylene fiber can be 0.5%, which is significant for cost saving in practical engineering. Wang et al. [46] showed that the optimal content of carbon black nanoparticles was 0.75 wt.%. That is, when the content of carbon black nanoparticles was less than the optimal content (less than 0.75 wt.%), cement-based composites’ compressive strength increased significantly with the content of carbon black nanoparticles. Tian [47] from Chongqing University found that with the increase in fiber content, the operating performance of the mixture was deteriorating, the fiber was obviously aggregated inside the fiber, and the molding was complex, which had a significant impact on the mechanical properties of the specimen. When the fiber content is between 1.5% and 2.5%, the overall compressive strength of cement-based materials will increase. However, when the fiber content is more significant than 2.5%, the compressive strength of cement-based materials will decrease significantly due to the limitations of mixing equipment and technology. Dai [48] conducted an in-depth study on steel fiber and concluded that steel fiber could only promote cementitious compressive strength within an appropriate range. The addition of steel fiber will curb the transverse deformation of the cement base under external load, which will slow down the destruction process of materials and thus improve the strength of cement mortar. However, after a certain amount of steel fiber is exceeded, Because the fiber has a specific volume, the frame of the mortar material loses its stability. The optimal dosage of this experiment is 1.5%, as shown in Figure 4. The influence of fiber content on compressive strength can be divided into two categories: The first is that with the increase of fiber, the compressive strength first increases and then decreases, as shown in the figure of PP fiber. This is because when the specimen is subjected to vertical load, the compression specimen is complete, and the bearing surface drops slightly. It does not break, resulting in a slight transverse displacement of the specimen. A small amount of fiber will limit the transverse displacement of the specimen, thus sharing part of the axial pressure, thus improving the specimen’s compressive strength. However, when the fiber continues to increase and exceeds the optimal content, the fiber will appear to be a clumping phenomenon, resulting in uneven fiber distribution. Moreover, the increase in fiber content will lead to increased bubbles introduced. The increased gas content in the specimen will hurt the compactness, thus reducing the compressive strength. The second is that the compressive strength gradually decreases with the increase of fiber. It can be explained that the positive effect covers the negative effect on ECC due to the significant increase of fiber, which makes the overall compressive strength decline.

The influence of different fibers on the compressive strength of ECC is different. Domestic and foreign scholars mainly use PVA fibers to conduct experiments on the performance of ECC. Therefore, PVA fiber-reinforced cement base is discussed without special instructions. 

### 2.2. Water-Glue Ratio of PVA-ECC

The water-binder ratio is an essential parameter of the concrete mix ratio, and the mechanical properties of concrete are directly related to the water-binder ratio. Under the condition that the variety, quality, and dosage of mixed materials remain unchanged, the water-binder ratio directly determines the strength of concrete. Therefore, scholars conducted a uniaxial compression test by changing the water-binder ratio to study the influence of the water-binder ratio on compressive strength. In the ECC compression test, Wang et al. [49] found that when the cement and fly ash content were the same, the larger the water-binder ratio was, the lower the compressive strength of the cube was. The smaller the water-binder ratio, the higher the compressive strength of the cube. Zhang et al. [50] analyzed that when the water-binder ratio is between 0.25 and 0.45, the compressive strength has almost a linear relationship with the water-binder ratio and decreases with the increase of the water-binder ratio. When the water-binder ratio is low, the water-binder ratio is between 0.26 and 0.30. The compressive strength changes more obviously with the water-binder ratio. Therefore, the influence of the water-binder ratio on the compressive strength increases when the water-binder ratio is low. This analysis result is the same as Gong [51], who made a comparative study on the influence of the water-cement ratio on PVA-ECC by comparing nine groups of different water-cement ratio test pieces with those made with traditional water-cement ratio. It found that the compressive strength of low-shrinkage PVA-ECC decreases with the water-cement ratio increase.

However, Tian [47] adopted the 0.25, 0.3, 0.35, 0.4, 0.45, 0.5, 6 groups of mixing water cement ratio up to 28 d, and found that the increase of water cement ratio for fiber reinforced cement base material mixture of compressive strength are presented first increases and then the influence of the phenomenon. Zheng [52] also obtained the same experimental results. When the fly ash content is constant, the compressive strength first increases and then decreases with the increase of the water-binder ratio. Kuang et al. [41] also conducted the water-binder ratio experiment. They tested the compressive strength of three groups of specimens with the water-binder ratio of 0.26, 0.30, and 0.34, respectively. The data analysis found that with the decrease of the water-binder ratio, the ultimate load and compressive strength were significantly increased, which reflected that the decrease of the water-binder ratio could improve the flexural and compressive strength with little ductility loss. Therefore, it can be concluded from their experiments that reducing the water-binder ratio is an effective way to improve compressive strength.

**Figure 5:**
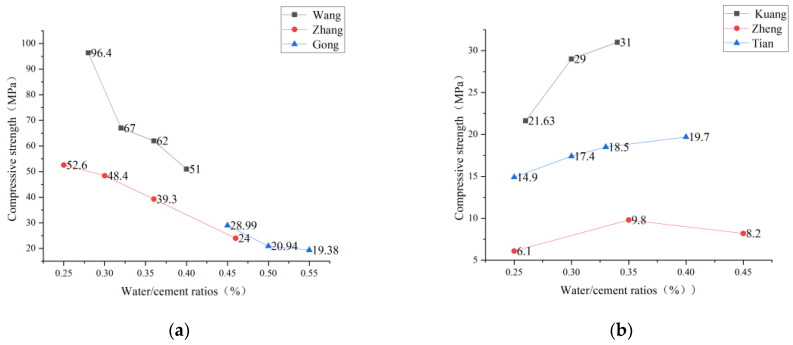
(**a**) Compressive strength of different water-glue ratios [41,50,51]. (**b**) Compressive strength of different water-glue ratios [41,47,52].

It can be observed from Figure 5. (a) below that the compressive strength gradually decreases with the increase of the water-adhesive ratio, while the following Figure 5. (b) shows that with the increase of the water-adhesive ratio, the compressive strength first increases and then decreases. The mechanisms behavior of water binder ratio [47] is that with the increase of the water binder ratio, the relative proportion of cement becomes smaller, and the pores left by the sand and gravel aggregate cannot be filled, resulting in large porosity, condense cement base and gradual decrease in compressive strength. The reason [51] is that when the water-binder ratio is low, the porosity inside the material is low and relatively dense. As a result, the compressive strength is high, but the lower water binder ratio could be more conducive to fiber dispersion and easy to appear agglomeration. Similarly, the fiber does not positively affect the matrix after cracking. The flexural strength is low, and the ductility is poor. With the increase in the water-binder ratio, the internal compactness of the system decreases, resulting in a decrease in compressive strength. However, the relatively loose internal structure and uniformly dispersed fibers make the fibers and cement matrix better combined, and the material’s flexural strength is improved to a certain extent. However, too large a water binder ratio will reduce the compressive strength and weaken the friction resistance between the fiber and the matrix, making the fiber easier to slide and pull so that the material’s flexural strength is reduced. Therefore, it is essential to find the best water binder ratio when preparing ECC test pieces.

### 2.3. Type of Mineral Admixtures

ECC matrix materials are mainly made of cement, quartz sand, auxiliary cementitious materials, and fiber as reinforcement materials and are added into the admixture to achieve ultra-high toughness. Many researchers have improved ECC in recent years and designed cement-based composites with excellent properties for economic or environmental protection. It is divided into two categories: ① Various mineral admixtures are used to replace cement in ECC raw materials; ② Recycled micro powder is used to replace quartz sand in ECC material.

**Figure 6:**
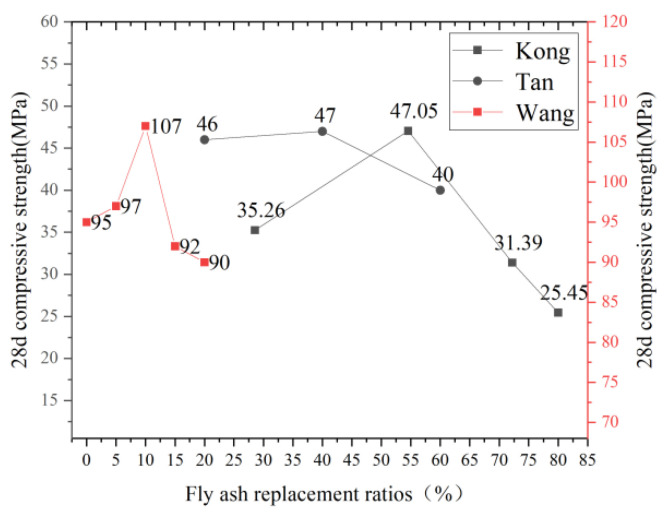
ECC compressive strength under different fly ash blending amounts [53,54,55].

As for the use of mineral admixtures to replace cement in ECC raw materials, the mineral admixtures used by domestic and foreign researchers mainly focus on fly ash, silica fume, and various solid waste powders. When fly ash replaces cement with different contents, the conclusions on its influence on ECC compressive strength are consistent. Wang [53], Tan et al. [54], and Kong et al. [55] found that within a specific range, fly ash replaces cement, and it has a positive effect on ECC compressive strength, which can be improved by about 20–30%. However, once the content exceeds a particular limit value, as the replacement rate of fly ash increases, ECC’s compressive strength will rapidly decline, as shown in Figure 6. When the fly ash content is not high, the cement is hydrated first to produce Ca (OH)_2_ and other substances. A large number of active ingredients SiO_2_ and Al_2_O_3_ in the fly ash will have a secondary hydration reaction with Ca (OH)_2_ to produce calcium aluminate hydrate and other substances, which fill in the gap of cement hydration products, enhancing the compactness of ECC and thus improving its strength. When the fly ash content is significant, the incorporation of fly ash reduces the cement clinker in the whole material, and the degree of hydration reaction of fly ash is lower than that of cement, so it cannot play the active certification role. Therefore, high fly ash content will reduce the strength of ECC, and the higher the fly ash content, the more noticeable this phenomenon is. Turk et al. [56] studied the effect of large-volume fly ash (FA) replacing cement (PC) on the durability of sustainable ECC. The ECC hybrid design included FA/PC ratios 1.2, 2.2, and 3.2. The experimental results showed that ECC specimens’ compressive and bending strength decreased with the FA/PC ratio increase. When FA/PC ratio is 3.2, the compressive strength is the lowest, 38.13 MPa.

**Figure 7:**
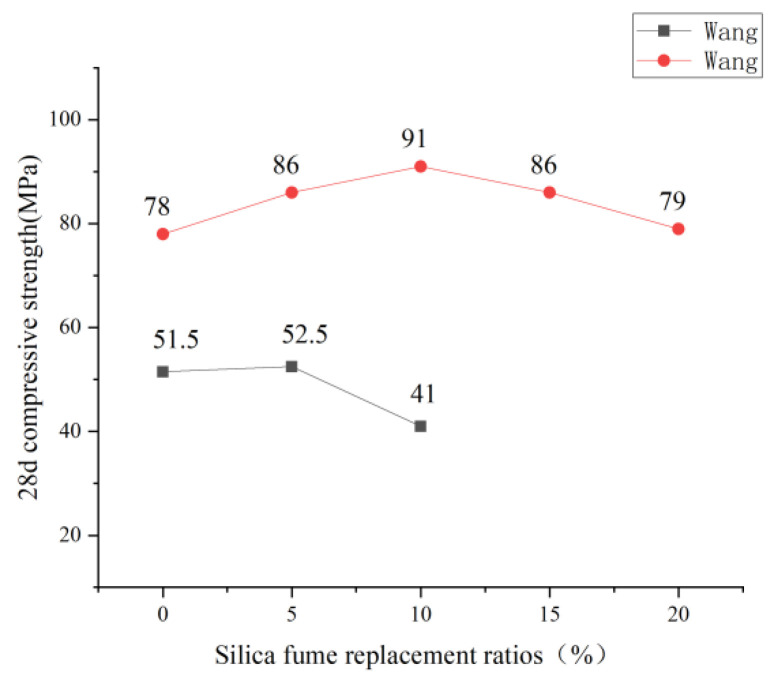
ECC compressive strength under different silica ash blending amounts [53,57].

**Figure 8:**
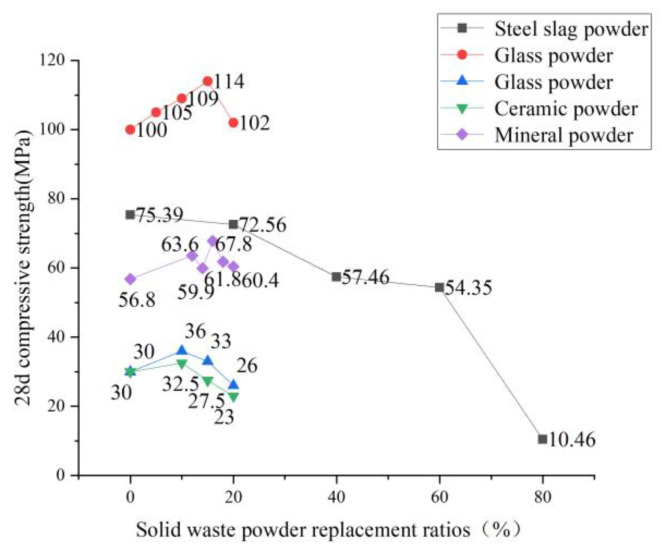
ECC compressive strength under different solid waste powder mixing amounts [53,58,59,60].

Silica fume replaces cement in ECC with different substitution rates, and its influence on compressive strength is consistent with fly ash replacing cement. See Figure 7 for specific trends. Silica fume is an excellent particle (0.01–0.3 μm) admixture. The size is much smaller than cement, through filling and “volcanic ash reaction” to make the internal matrix pores fine, effectively increasing the density of the cube test block to improve the compressive strength. However, volcanic ash’s reaction still requires cement hydrate participation. Therefore, when the content of silica fume reaches a certain level, the reaction of volcanic ash is insufficient, resulting in a decrease in the compressive strength of ECC. All kinds of solid waste powder partially replace the cement of ECC raw materials. The degree of influence on ECC’s compressive strength varies, but the overall trend is the same. Under a small dosage, it has little influence on the compressive strength of ECC, and the strengthening or weakening is controlled within 20%. However, after a specific dosage is exceeded, the compressive strength of ECC will decrease significantly. See Figure 8 for the specific trend. Wang [53] studied the influence of different kinds of auxiliary cementing materials on the mechanical properties of ECC and found that when glass powder replaced a small amount of cement, it positively impacted the compressive strength of ECC. Wang [57] also used glass powder instead of cement, and the result was the same as before, which had a slight positive impact on the compressive performance of ECC. Xiao [58] used steel slag powder as cementing material to prepare ECC, and slag powder content was as follows in order of gradient: 0%, 20%, 40%, 60%, and 80%. The mechanical properties of ECC were tested through a series of tests, such as cubic compression. The research found that with the increase of slag powder content, its compressive strength gradually decreased, and the higher the content, the more significantly the strength declined. Zhou [59] added 0%, 12%, 14%, 16%, 18%, and 20% mineral powder. The test found that the strength of the material showed a trend of rising and then decreasing. Yan [60] also experimented with mineral powder, and obtained the results that the compressive performance was first enhanced and then decreased. The specific trend is shown in Figure 8. Solid waste powder plays its micro-aggregate filling role. It forms a gradually changing transition layer in the interfacial transition zone, which improves the weak link of the interface between cement slurry and aggregate. The matrix’s harmful pores are reduced, and compactness is improved, which is conducive to the strength and durability of the material [61]. In the grinding process, the solid waste powder produces cracks in the powder particles, resulting in the original defects of the solid waste powder. Moreover, incorporating the solid waste powder reduces the cement content in the matrix. Therefore, exceeding a specific dosage will negatively impact ECC’s compressive strength once a specific dosage is exceeded. Therefore, due to the consideration of ECC compressive strength, the dosage of mineral admixture instead of cement in ECC should not be too large.

As for replacing quartz sand in ECC material with recycled micro powder, domestic and foreign researchers mainly focus on two kinds: recycled brick powder and recycled concrete powder. Li et al. [62] studied the compressive strength of different recycled concrete powder contents and sizes. Compared with the control group without recycled concrete powder, 20% of 0–300 μm recycled concrete powder ECC was added. Maintain the same level of compressive strength. Cheng et al. [63] replaced quartz sand with recycled brick powder with replacement rates (0%, 25%, 50%, 75%, and 100%), respectively, and studied the ECC compressive strength of recycled brick powder. The results showed that the compressive strength of quartz sand after being entirely replaced by recycled brick powder only decreased by 10% compared with the control group. It is also explained that although the recycled brick powder has similar fineness compared with quartz sand, it has lower density and strength, so its compressive strength decreases with the increase of replacement rate. Yu et al. [64] replaced quartz sand in ECC materials with recycled micro powder. They explored the influence of replacement rates of 50% and 100% recycled micro powder on the performance of ECC materials. The research results showed that the compressive strength of recycled micro powder ECC showed a trend of rising and decreasing with the increase of recycled micro powder content. However, the compressive strength of ECC can be improved no matter how much the recycled micro powder replaces quartz sand. Shi [65] studied that 25%, 50%, 75%, and 100% quartz sand was replaced by the mixture of recycled concrete powder and brick powder, and the compressive strength of the matrix increased with the increase of the proportion of concrete powder. Therefore, the regenerated micro powder can replace quartz sand to prepare ECC.

## 3. Tensile Strength

The advantage of ECC material performance is mainly reflected in its tensile performance, with prominent stress-strain characteristics. Because the uniaxial tensile experiment is simple to operate, has low requirements on test instruments, and can effectively reduce the influence of eccentric tension on test results, high success rate, and accurate test results. The uniaxial tensile tests are used to evaluate the unique mechanical properties of ECC when studying tensile strength.

### 3.1. Type of Fiber

Nowadays, the tensile strength of PVA fiber, P.P. fiber, basalt fiber, and carbon fiber mixed with ECC is mainly studied at home and abroad. Different types of fiber have different tensile properties. Yao et al. [66] tested the tensile properties of P.P. fiber-cement base with a fiber content of 0.5 % (volume fraction, same below) and 1.0 % by conducting the splitting tensile test. Considering the two failure mechanisms in the splitting test [67], they found that the tensile strength of P.P. fiber specimens with different mixing amounts was significantly improved. However, the tensile strength decreased at 28 days. Yao and Li et al. [68] studied PP-ECC. They concluded that with the growth of age, the number and volume rate of open holes in concrete significantly decreased, and the number of closed holes increased sharply, resulting in a decreasing trend of tensile strength at 28d. As a new filamentous mineral additive, basalt fiber has been studied by domestic and foreign scholars because of its low cost. Zhu [69] found that the tensile strength of basalt fiber fabric was low, and the tensile strength could be significantly increased with the addition of staple carbon fiber, glass fiber, and steel fiber. It would continue to increase with the increase in staple fiber’s volume content. The tensile strength reached the maximum when the volume content was 1.5%. Dimas [70], a foreign scholar, explored the influence of high temperature on basalt fiber-reinforced calcium aluminate cement-based composites and concluded that the tensile strength of the composites under residual conditions was higher than that under hot conditions. The increase in temperature decreased the tensile strength, elastic modulus, and strain capacity. Wang et al. [71] find that the BF-ECC splitting tensile strength increases and decreases with basalt fiber content and length according to the splitting tensile test. Maalej et al. [72] studied the reinforcement effect of Steel fiber on ECC. Due to the high elastic modulus of steel fiber but the trim length and diameter ratio, the steel-ECC specimen prepared with steel fiber had higher ultimate tensile strength but lower tensile strain. Therefore, the characteristics of high elastic modulus and low price of steel fiber can be fully advantaged in the research process. The P.E. and PVA fibers with low elastic modulus and high price can be used together. SOE et al. [73] developed a new hybrid fiber reinforcement system, which can achieve higher tensile strength and tensile strain while reducing ECC cost. Due to the high strength and modulus of steel fiber, it can improve the tensile strength of steel fiber, while PVA fiber shows significant improvement in mechanical properties. Moreover, it can create molecular bonds to improve ductility and tensile strength significantly in the dehydration process, so hybrid fiber has a vast application prospect. Chinese scholar Wang et al. [74] compared the experimental conditions of various fibers: ECC specimens made of carbon fiber and basalt fiber had a higher cracking load but poor ductility. ECC specimens prepared by PVA fiber and P.P. fiber have a relatively low cracking load but good ductility, showing a multi-point cracking phenomenon during failure. ECC specimens prepared by P.P. fiber have tiny cracks and a relatively wide maximum crack width. In contrast, ECC specimens prepared by PVA fiber have a large number of cracks and a relatively small maximum crack width. Based on the comprehensive strength, ductility, strain hardening index, and toughness index, domestic PVAF2 and P.P. fibers can be considered for preparing ECC, replacing the expensive Japanese PVA fibers.

Studies on factors influencing the tensile properties of ECC by domestic and foreign scholars mainly focus on the experiments of PVA fiber. Therefore, studies are conducted on PVA fiber-reinforced cement base without particular explanation below. 

### 3.2. Content of PVA Fiber

**Figure 9:**
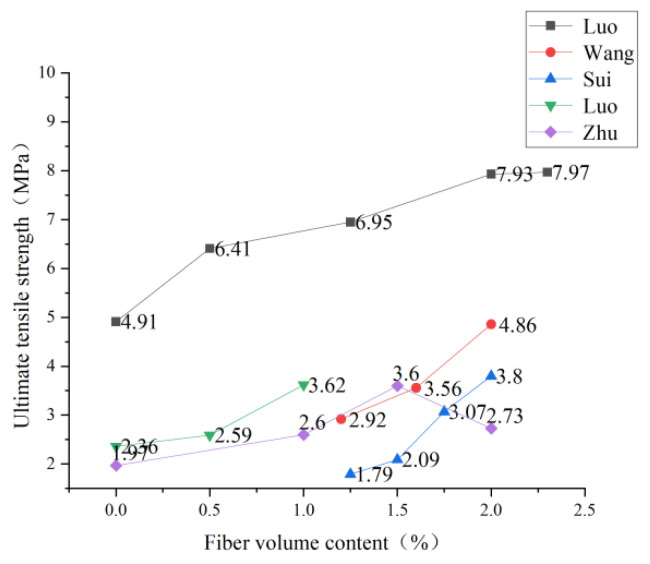
Fiber admixture and ultimate tensile strength [65,75,76,77,78].

**Figure 10:**
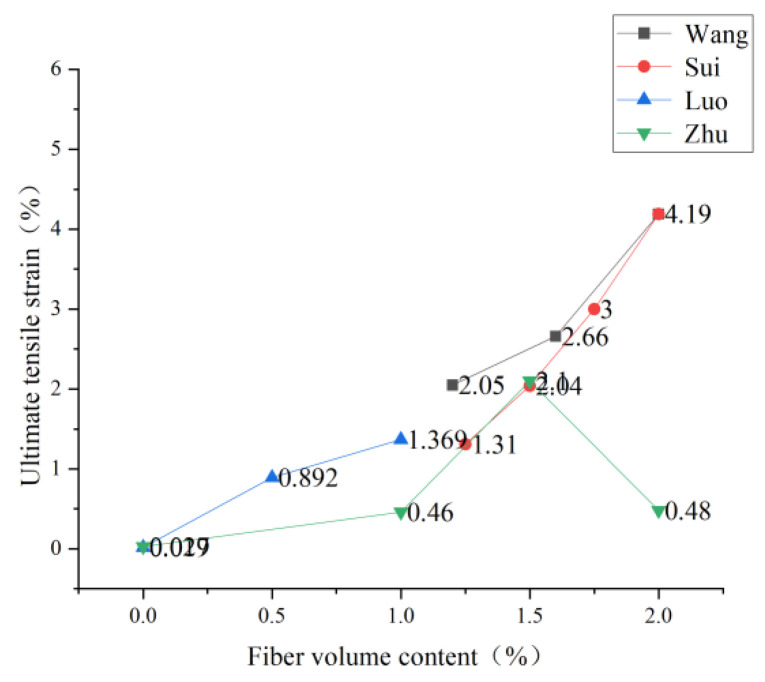
Fiber admixture and ultimate tensile strain [65,76,77,78].

The PVA dosage will directly affect ECC’s tensile strength, and studying the optimal dosage helps reduce the cost and enhance performance. Luo et al. [75] found through the static splitting tensile test that the static splitting strength increased with fiber content and reached the maximum splitting strength of 7.7 MPa when the fiber content was 2.3%. However, there was a threshold value for the strengthening effect, and there would be no significant change after that. Zhu et al. [76] found that due to the crack resistance effect and bridging effect of PVA fiber, the cracking strength, cracking strain, ultimate tensile strength, and ultimate tensile strain of ECC thin plate, all increased with the increase of PVA fiber volume content. When the volume of PVA fiber is 2.0%, the bridging effect of FRP fiber can be fully utilized, and the tensile performance of ECC is also the most significant improvement. Sui [65] also obtained the same result as Zhu in the experiment. When the volume content of PVA fiber reaches 2%, its tensile strain-hardening characteristics are the most obvious, and the specific trend is shown in Figure 9. After adding a large amount of PVA fiber to the ECC matrix, the fiber inhibited the generation of cracks before cracking, and the fiber further inhibited the crack propagation after cracking. The bridging stress between the fiber and the matrix resulted in more micro-cracks in ECO-ECC, which significantly enhanced the ductility of ECC and thus increased the ultimate tensile strength. Luo [77] studied the failure form of PVA-ECC. With the increase in fiber content, the failure mode of PVA-ECC changes. When the fiber content is 0, brittle failure occurs. When the fiber content is 0.5%, the ductility is significantly improved. When the fiber content is 1%, a typical strain hardening phenomenon appears, and the ductility is further improved. The specific trend is shown in Figure 10. With the increase of the fiber content, the ultimate tensile strain increases because the gradual addition of fibers into the ECC matrix will strengthen the bridging effect between the fibers and the matrix, thus restraining the generation of cracks and enhancing the ductility of ECC. Wang [78] found that the increase in fiber content helped improve the seismic performance of pier columns, which further expanded the application prospect of the fiber-reinforced cement base.

### 3.3. Water-Binder Ratio

**Figure 11:**
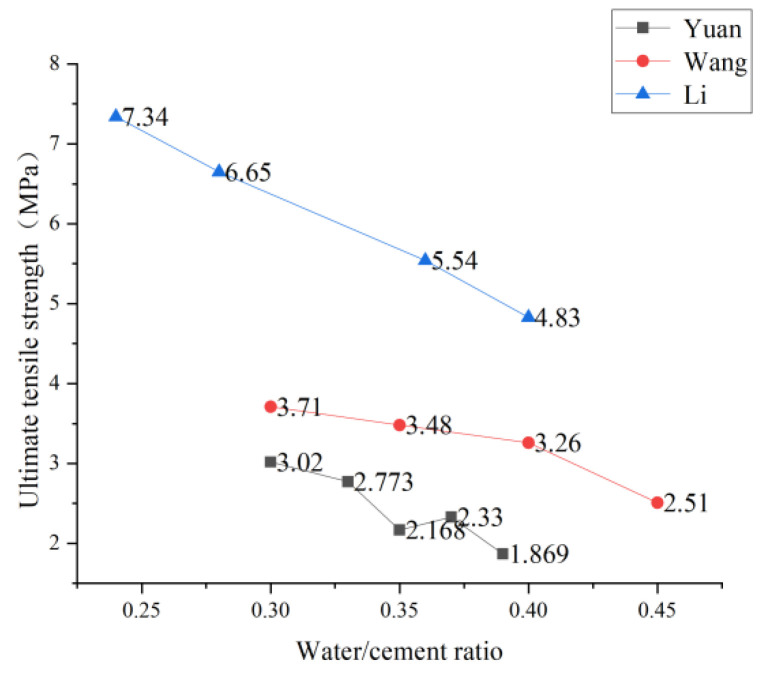
Relationship between water-binder ratio and ultimate tensile strength [80,81,82].

**Figure 12:**
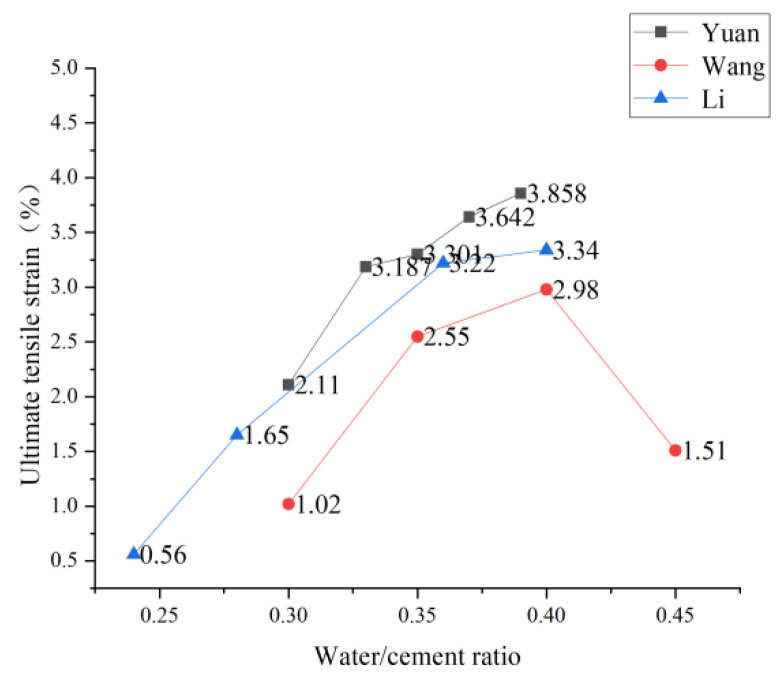
Relationship between water-binder ratio and ultimate tensile strain [80,81,82].

Theoretically, the increase in the water-cement ratio will reduce the strength of the cement base and, thus, the tensile strength of PVA-ECC [79]. However, Kuang [41], Yuan et al., and Li et al. conducted uniaxial tensile experiments by changing the water-cement ratio and found that the lower the water-cement ratio is not, the better. However, in an appropriate range, PVA-ECC has the best tensile performance. Kuang [41] experimented on the equivalent ultimate tensile strain and found that the equivalent ultimate tensile strain at the water-cement ratio of 0.35 was more significant than that at the water-cement ratio of 0.21. In contrast, the equivalent ultimate tensile strain at the water-cement ratio of 0.28 was more significant than that at the water-cement ratio of 0.35, indicating that there was no simple linear relationship between the water-cement ratio and the tensile strength. Yuan et al. [80] conducted many experiments on the water-binder ratio of recycled brick powder ECC. They found that the ultimate strain of the material increased with the increase of the water-binder ratio, and the ultimate stress showed an overall downward trend with the increase of the water-binder ratio. When the water-binder ratio was 0.37, recycled brick powder’s ECC bending and uniaxial tensile properties were the best. When Li et al. [81] increased the water-binder ratio from 0.24 to 0.28, 0.32, 0.36, and 0.40, respectively, it was observed that with the increase of water-binder ratio, the ultimate tensile strength of PVA-ECC at 7 and 28 days decreased, and the specific trend was shown in Figure 11. However, the tensile strain hardening and ductility of PVA-ECC with a water-binder ratio of 0.40 were significantly improved. The ultimate tensile strain at 28 days was 5.96 times that at a water-binder ratio of 0.24. After changing the water-binder ratio and conducting many uniaxial tensile tests, Wang et al. [82] believed that when the water-binder ratio was appropriate. PVA-ECC could fully realize multi-point cracking, and the ultimate tensile strain could reach more than 3.0% and up to 5.7%. The specific trend is shown in Figure 12.

Based on the research of many scholars, the relationship between the change of effluent glue ratio, ultimate tensile strength, and ultimate tensile strain can be obtained, as shown in Figure 11 and Figure 12. Therefore, a low water-binder ratio is conducive to the dispersion of PVA fibers. However, a low water-binder ratio will increase the fracture toughness of the matrix, which is not conducive to the play of strain hardening characteristics. In addition, a low water-binder ratio will also reduce the workability of fiber-reinforced cement-based composites, which is not conducive to typical construction. In contrast, a high water-binder ratio will increase the porosity of the matrix and reduce the elastic modulus of the matrix. Therefore, an appropriate range of water-binder ratio should be selected in the preparation of PVA-ECC.

### 3.4. Type of Mineral Admixtures

**Figure 13:**
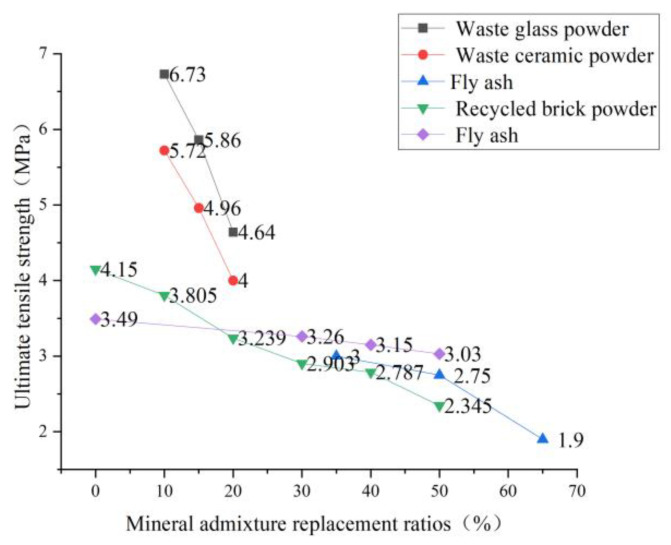
Mineral admixture mixture quantity and ultimate tensile strength [1,57,60,86].

**Figure 14:**
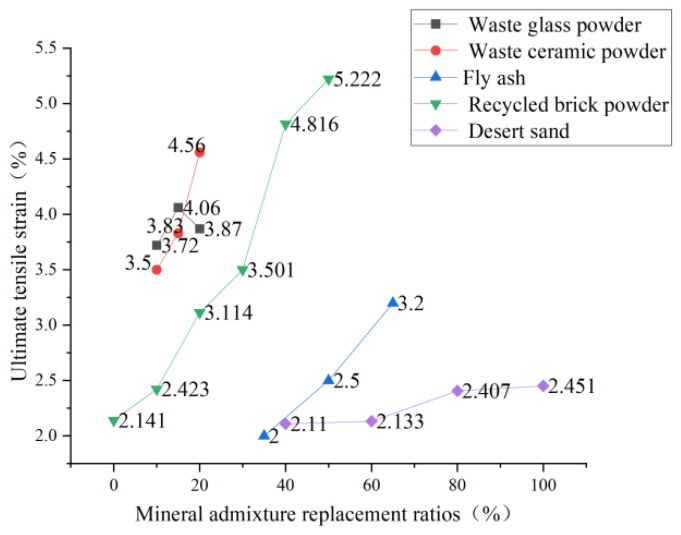
Mineral admixture mixture quantity and ultimate tensile strain [1,57,60,89].

In recent years, Li et al. [83] have been exploring slag admixture to replace part of cement as cementing material and have successfully produced fiber-reinforced alkali-excited cement-based composite material. This material reduces the amount of cement, is more environmentally friendly than traditional ECC, and can also obtain an excellent strain-hardening effect. Specimens’ uniaxial tensile test results show that the ultimate tensile strain can reach 4.48%, which is close to the tensile strain level of ECC members [84]. Zhou [85] concluded that by making full use of the “secondary hydration” effect of industrial by-products and applying it to the preparation of ECC, the purpose of environmental protection, waste benefit, and cost reduction can be achieved at the same time. Yan [60] replaced cement with waste glass powder and ceramic powder, respectively, for the uniaxial tensile test and found that the ultimate tensile strain of the specimen increased with the increase of waste solid material content. When waste glass powder was added, the ultimate tensile strain increased from 3.29% to 4.1%. Zhou [24] replaced cement with silica fume to study the variation of its tensile strength and found the same variation rule. Chen [86] and Wang [57] from Southeast University used fly ash instead of cement in the experiment and found that the tensile strength decreased with the increase of fly ash. Guo et al. [87] designed an orthogonal test and found that the order of influence on tensile strength was: water-binder ratio > PVA fiber content > recycled micro-powder content > water-reducing agent > sand-binder ratio. The tensile strength was the highest when the recycled micro-powder content was 25%. The tensile strength decreased with the increase in the content. In the field, the recycled brick powder was used to replace part of the cement, and the recycled brick powder -ECC specimen was prepared for the uniaxial tensile test [88]. It was found that with the increase in the recycled brick powder replacement rate, the initial crack strength and initial crack strain of RBP-ECC showed a downward trend, and the matrix strength of RBP-ECC decreased. The stress transmitted by the cracked fibers to the surrounding cement matrix is more likely to make it crack. Che et al. [89] replaced quartz sand with desert sand. They found that desert sand improved the PVA/matrix interface and aggregate/matrix interface characteristics, which were conducive to fiber pull-out slip and improved the late tensile strength of materials. The experimental data of many scholars on mineral admixture are collected in Figure 13 and Figure 14. When mineral admixture is added to ECC, the ultimate tensile strength decreases, but the ultimate tensile strain increases with the dosage increase. It can be explained that adding mineral admixture improves the compactness of the cement matrix, reduces the porosity, and increases the strength of the matrix. The matrix is not easy to crack during the tensile process, which makes the PVA-ECC obtain the tensile ductility to maintain the long-term tensile strain. However, the current research level needs to be more profound, and the experimental data needs to be improved. The research scope should be expanded in the future, which will also have long-term significance for saving costs and reducing municipal solid waste.

Generally, when studying the compressive strength of engineering fiber cementitious composite materials, scholars adopt the uniaxial compressive test method, and when studying tensile strength, the uniaxial tensile test is adopted. They all establish linear models through fitting to conclude mechanical properties. However, the final applications are different due to the variety of composite materials. For the engineering fiber cementitious composite mixed with different fibers, although the tensile strength of PVA fiber is not as high as PE fiber, its price is much lower than PE fiber on the premise that it can meet the requirements of ECC design theory on fiber. Therefore, in recent years, PVA fiber has gradually replaced PE fiber. More and more people are studying PVA-ECC in China. Another example is a mixed fiber-reinforced cement-based composite material. It can make up for the shortcomings of a single fiber to have better all-around performance. Under the premise of meeting the requirements of use, we can consider the fiber cost, environmental protection, and other factors. Therefore, composite materials can be more practical, and scholars focus on mixed fiber cement composites.

Mineral admixtures include fly ash, silica fume, and a variety of solid waste powders. Fly ash and silica fume have the same characteristics when they substitute cement into ECC. Therefore, increasing the substitution rate has positive effects within a specific range. Considering the compressive strength of ECC, the dosage of mineral admixture instead of cement in ECC should be a manageable size [56].

Therefore, the difference in composite materials gives the application range its characteristics. In practical life, the application of composite materials should be determined according to the specific practical needs.

## 4. Bending Strength

ECC has prominent strain-hardening properties under bending loads [90]. In recent years, strain-hardening cement-based composites have been widely used as an advanced building material. Due to the simple operation of the four-point bending test, low requirements on test instruments, high success rate, and accurate test results [91], the four-point bending test is used to evaluate the unique mechanical properties of ECC in most studies of bending strength.

### 4.1. Type of Fiber

The flexural strength of different fiber types will change after the addition of ECC. Scholars at home and abroad have mixed different kinds of fiber into ECC and measured its flexural strength by a four-point bending experiment. Liu et al. [92] found that the flexure strength of PVA-ECC increased with the increase of PVA fiber content. At the same time, the increase of PVA fiber content could significantly improve the ultimate bending tensile strain of specimens, and the optimal content was 1.5% of PVA fiber volume. Xu et al. [93] conducted experiments with various fibers and found that the ECC initial crack deflection of single-doped fiber was proportional to the fiber content. Under the same content, the initial crack load of PP fiber corresponds to the most significant bending deflection, followed by steel fiber and the smallest PVA. Maalej et al. [94] studied the flexural properties of strain-hardened engineering cementing composites (ECC) and compared them with those of conventional fiber-reinforced cementing composites (FRC). They found that in the third point bending test, ECC’s bending strength was measured five times its tensile strength (the first layer cracking). Kamile et al. [95] applied plasma-treated PE fiber as reinforced fiber and found that the bending strength and toughness were improved through the bending test. Based on the above studies, it is concluded that the flexural strength of fiber-reinforced cement base is much higher than that of the ordinary cement base. All kinds of fibers can improve flexural strength, but the degree of improvement is different. According to the function of ECC in practical engineering, selecting the appropriate fiber into the matrix will improve the actual engineering effect. Since the research on the bending strength of ECC is mainly focused on PVA-ECC, the following are all studies on PVA-ECC.

### 4.2. Content of Fiber

**Figure 15:**
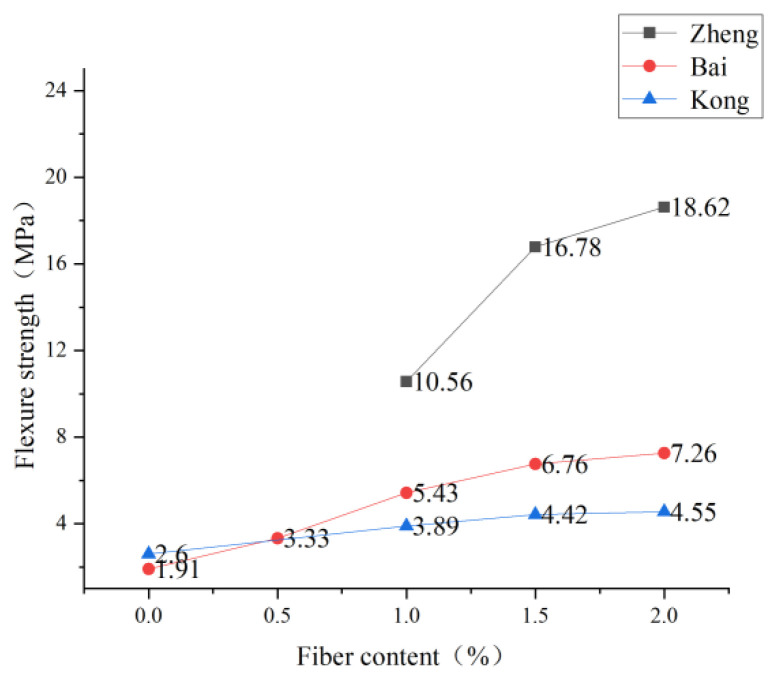
Fiber incorporation and flexural strength relationship [55,96,97].

Generally, the minimum fiber volume to maintain strain hardening is called the critical volume. The ECC cannot have good bending strength if the fiber content is lower than the critical volume. Due to the high price of fiber, it is feasible to reduce fiber content at the expense of ECC toughness to a certain extent in some specific situations. Zheng et al. [96] carried out four-point bending tests on PVA-ECC with volume content of 1.0%, 1.5%, and 2.0%, respectively, when the water-binder ratio and mineral admixture were fixed and found that the bending strength increased with the increase of fiber admixture. See Figure 15 for the specific trend. Bai et al. [97] found a similar situation. With the increase in PVA fiber content, the bending strength of the ECC specimen increased significantly. After adding 2.0% PVA fiber, the bending strength of the ECC specimen was 3.8 times that of the corresponding matrix specimen because the addition of PVA fiber could limit the development of tensile cracks and improve the bending strength of ECC material. Kong et al. [55] found that with the increase of PVA fiber volume content (1.0%→2.0%), the toughness index of ECC first increased and then decreased, reaching the maximum at 1.5%. Therefore, increasing PVA fiber content could improve the initial crack strength and strain hardening effect when the first crack appeared. Li [13] deduced the strength and strain of the crack from the micromechanical properties of fiber, matrix, and interface and also found that fiber content greatly influenced the appearance of the first crack. Thus, the bending strength will increase with fiber content. An appropriate fiber content increase will increase ECC’s flexural strength if the economy permits.

### 4.3. Water-Binder Ratio

**Figure 16:**
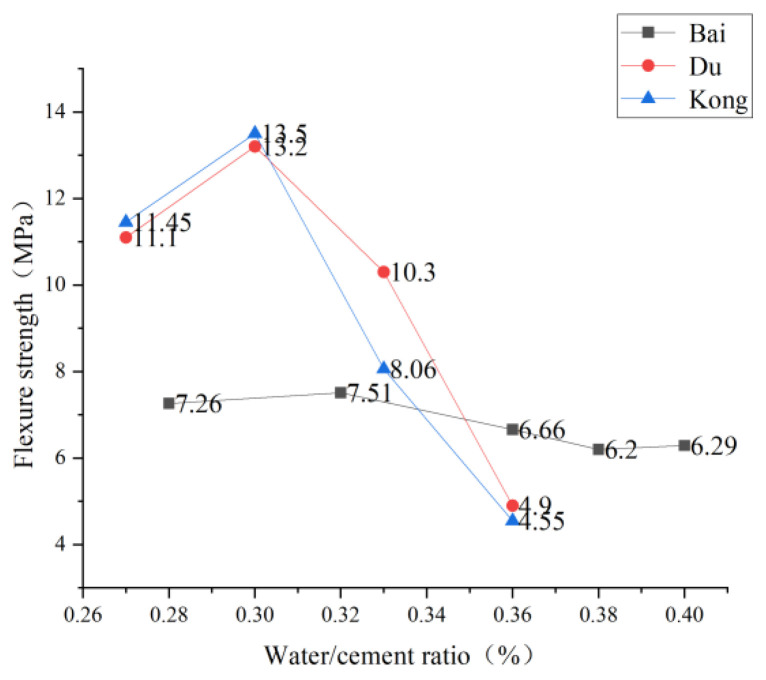
Effect of water glue ratio on flexural strength [55,97,98].

According to the four-point bending experimental data, Du [98] found that ECC’s strength decreased with the water glue ratio. When the water glue ratio increased from 0.27 to 0.30, 0.033, and 0.36, the bending strength of ECC increased by 17.90% first and then decreased by 9.17% and 60.26%, respectively. Through this experiment, Kuang [41] concluded that with the decrease of the water glue ratio, the maximum deflection of the specimen decreases slightly, and the limit load is significantly increased. Reducing the water-glue ratio can improve the bending strength when the ductility loss is insignificant. Based on the four-point bending test of a thin plate, Kong et al. [55] concluded that the strength of the first crack of ECC mainly depends on the influence of the water-glue ratio. At the same time, the strain hardening and softening effects are mainly dominated by the amount of PVA fiber mixing. Cao [99] also found that the PVA-ECC with water glue ratio of 0.29, 0.35 and 0.40 was 4.47, 4.60 and 5.01 times that of matrix concrete, respectively, so the increase of water glue ratio improved the toughness of the material. Zheng [96] found that the cracking strength and tensile strength decreased with the increase of the water glue ratio because the increase of the water glue ratio reduced the strength of the matrix, and the stress decreased significantly after the water glue ratio was more significant than 0.4. As shown in the curves of Du L. and Kong Y., the bending strength changes sharply with the increase of the water glue ratio, and the downward curve of Bai et al. [97] is changed gently. Therefore, the more appropriate water glue ratio should be selected to prepare ECC to ensure the best bending performance and toughness at this time. The experimental data drawn in Figure 16 below shows the changing trend with the increase of water ash ratio; bending strength first increases and decreases. The reason is the low water glue ratio of PVA fiber disorderly distribution and deformation hardening characteristics. With the increased water glue ratio, the PVA fiber presents uniform distribution to improve the bending strength. A higher water glue ratio can easily cause poor adhesion and porosity when the matrix bending strength gradually reduces over a specific range. However, the high water-binder ratio can make the PVA fibers in the matrix’s relatively loose internal structure easy to pull out from the matrix, which is conducive to playing the ductility properties of PVA-ECC materials. Hence, the ECC ductility of the low water glue ratio is poor.

### 4.4. Amount of Fly Ash Mixed

**Figure 17:**
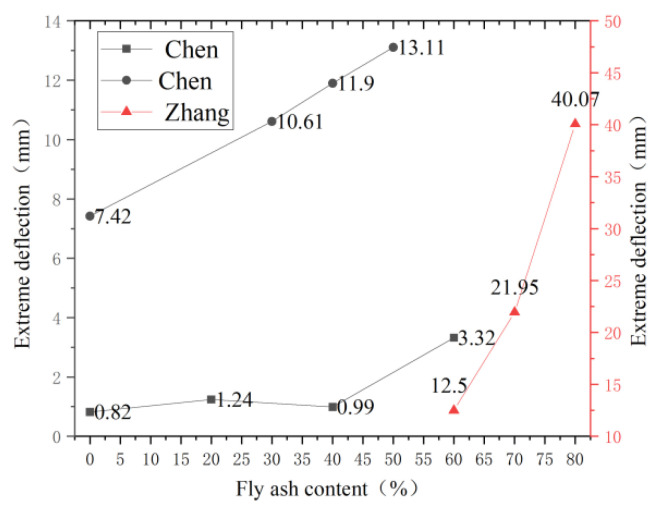
Fly ash blending and limit deflection [86,100,101].

Fly ash not only affects the compressive strength of ECC material but also significantly affects the bending toughness of ECC material. Chen [100] set the replacement cement dosage of fly ash as 0, 20%, 40%, and 60%, respectively, and found that the greater the fly ash content, the greater the ultimate deflection, but when the fly ash content was 40%, the ultimate deflection was only 0.99 mm. Zhang [101] found that the ultimate mid-span deflection reached its maximum value when the fly ash content was 80%, which increased by 28.6% and 225.6%, respectively. With the increase in fly ash content, the ductility of the PGVA-ECC was significantly improved compared with the fly ash content of 60%. Chen [86] analyzed through the four-point bending test results that the chemical adhesion and friction adhesion at the interface between PVA and matrix gradually decreased with the increase of fly ash. In contrast, the relative slip force between the fiber and matrix interface increased, resulting in the high toughness of PVA-ECC. The following figure shows the experimental data of the three persons. With the increase of fly ash content, the curves of the ultimate deflection all rise. The experimental results of Yang et al. [102] show that the long-term tensile ductility of HVFA concrete is about 2–3% when a large amount of recycled fly ash is added. According to the Figure 17, with the increase of fly ash content, the crack width and free drying shrinkage of the HVFA-ECC structure decreased significantly, which was beneficial to the long-term durability of the structure. In conclusion, appropriate fly ash can effectively improve the interface bonding force between PVA fiber and matrix and improve strain hardening characteristics. Therefore, with the increase in fly ash content, the bending strength of ECC specimens shows an increasing trend, and the bending toughness is constantly improved. Therefore, to meet the requirements of compressive strength, appropriately increasing the fly ash content is helpful to improve the bending toughness of ECC further.

## 5. Drying Shrinkage

Drying shrinkage is an inherent property of cement-based materials. In a dry environment, the internal humidity of cement-based materials is greater than the ambient humidity, resulting in water loss in the hydration phase. In an engineering environment where the relative humidity is greater than 40%, the capillary force and dismounting pressure caused by water loss cause the cement-based materials to shrink, and the drying shrinkage accounts for 80–90% of the total shrinkage [103]. Its 28-day drying shrinkage value can reach 1200–1800 µε, which is three times that of ordinary concrete at the same age [104]. Therefore, when ECC is used as a repair or connection material, it cannot work with the newly poured concrete or the old concrete surface. The interface damage occurs due to the disharmony of deformation, leading to the interface layering between the new and old materials [105]. Therefore, studying how to reduce the drying shrinkage of ECC will be conducive to the more comprehensive promotion and application of ECC. Since the current research on dry shrinkage mainly focuses on PVA-ECC, this paper uses PVA-ECC to study the influencing factors.

### 5.1. Water-Binder Ratio

**Figure 18:**
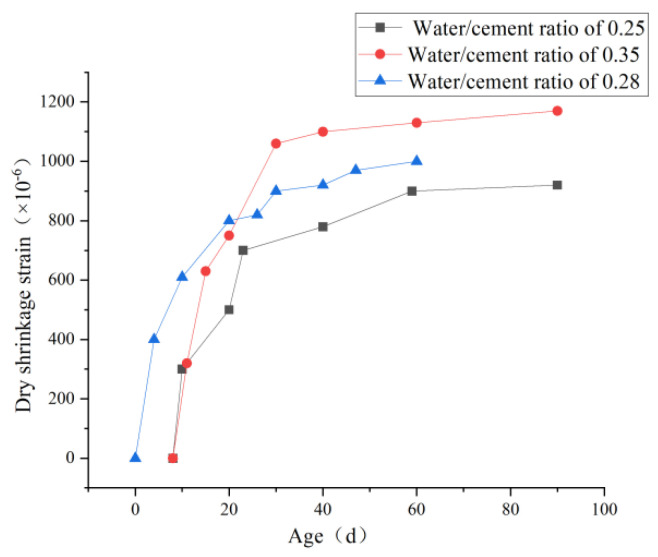
Dry shrinkage and water-binder ratio: [107,108].

The water-binder ratio is the proportion of each material in PVA-ECC, which affects the amount of hydration reaction and causes altered chemical contraction and self-contraction. For different water-binder ratios, the drying shrinkage of ECC increases with the water-binder ratio, and the smaller the water-binder ratio is, the more pronounced the increase is [106]. It is because the water content of the material is proportional to the water-binder ratio. Therefore, the material water content is small, the water lost during drying shrinkage decreases, and the shrinkage value decreases. By drying the contraction experiment of ECC specimens, Zhou et al. [107] found that the contraction strain had reached 80% of the total contraction at 20 d, and the later growth was stabilized at 40 d. When the water-glue ratio increased from 0.24 to 0.26 and 0.28, the contraction strain value increased by 10.4% and 20.6%, respectively. Wang et al. [108] found that the self-contraction ratio of 0.55, 0.50, and 0.45 decreased by 23.53%, 16.88%, and 25.07%, respectively. The self-contraction contraction of the low water-binder ratio was more significant than that when the water-binder ratio changed in a small range. The self-contraction of PVA-ECC increased with the increase of the water-binder ratio. Figure 18 shows that with age increase, the greater the self-contraction value and the greater the water-glue ratio, the more pronounced the self-contraction is. The water-binder ratio has two mechanisms of action on drying shrinkage. The first is that the water-binder ratio increases, the gel material used decreases, the hydration reaction amount decreases, and the self-contraction decreases. The second is that the water-binder ratio increases, the specimen density decreases, the capillary aperture increases, and the existence of PVA fiber also increases the material’s internal pores, enhancing water carrying and transportation, making it easier to migrate to the reaction area. Since the second effect is strong, the overall trend shows a more obvious self-contraction with an increasing water-binder ratio.

### 5.2. Content of Fiber

**Figure 19:**
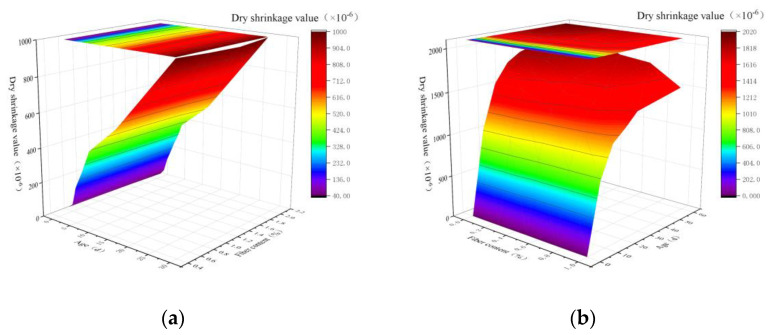
(**a**) the drying shrinkage of different fiber dosages [111]; (**b**) the drying shrinkage of different fiber dosages [112].

The fiber content affects the mechanical properties of ECC and has a subtle effect on its drying shrinkage. Meng et al. [109], a foreign scholar, found that PVA fiber could significantly reduce the autogenous shrinkage of the material. Later, Ma et al. [110] found that the autogenous shrinkage rate of high-performance concrete decreased with the increase in the content of carbon fiber. Miao et al. [111] conducted a drying shrinkage experiment by changing the content of PVA fiber. They found that with the increase in the content of PVA fiber, The shrinkage strain of PVA-ECC material decreases, as shown in Figure 19a. However, the reduction is limited, and the shrinkage strain of the sample with 2% fiber content decreases by 5.4% compared with that with 0.5% fiber content. It is because the PVA fibers in the material hinder the extension of microcracks caused by shrinkage in the substrate, and the PVA fibers in the cracks bear the shrinkage stress, thus reducing the shrinkage strain. Zhou [112] also conducted this experiment and reached the same conclusion, as shown in Figure 19b below. With the increase in PVA fiber content, cement-based composites showed a phenomenon of decreasing shrinkage strain. With the increase in fiber content, more fibers can constrain and block the expansion and extension of microcracks caused by shrinkage in specimens and bear part of the stress caused by matrix shrinkage, thus reducing the shrinkage strain of materials [113]. Therefore, appropriately increasing the fiber volume content of PVA in ECC helps reduce the drying shrinkage of ECC.

### 5.3. Amount of Fly Ash Mixed

**Figure 20:**
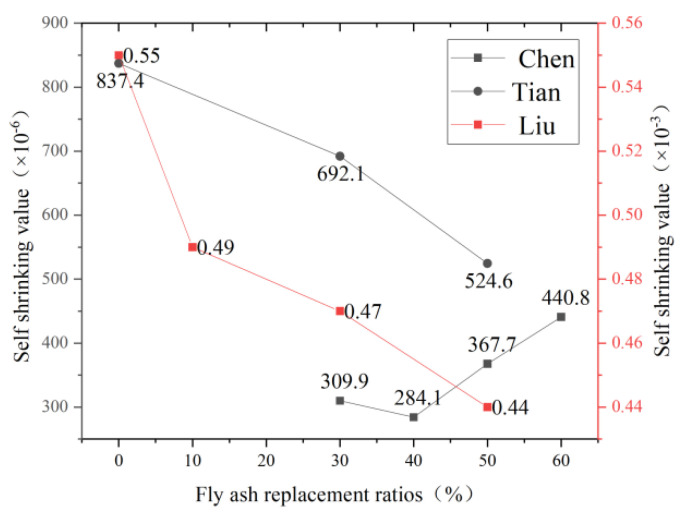
Fly Ash Mixed and Self-shrinking [114, 115, 116].

As a kind of volcanic ash mineral admixture, fly ash can effectively improve the durability of cement-based materials, and different fly ash admixtures have specific effects on the drying shrinkage of the cement base. Domestic and foreign scholars have conducted dry shrinkage experiments on ECC specimens with different dosages, but the experimental results are slightly different. Chen et al. [114] in China found that the best fly ash content was 40%. At 180 days, the dry shrinkage strain of ECC with fly ash content of 30%, 40%, and 50% decreased by 25.4%, 31.6%, and 12.4% compared with the group without fly ash, respectively, but when the fly ash content was 60%, the dry shrinkage strain increased by 6.2%. Liu et al. [115] also found that when fly ash content was 10%, 30%, and 50%, the final shrinkage value of concrete was reduced by 4.6%, 18.5%, and 35.2%, respectively, compared with that without fly ash, indicating that the inhibition effect on shrinkage was more significant when fly ash content was higher. Tian’s experiment [116] was different from the two. When the fly ash content increased from 30% to 40%, the self-shrinking strain decreased, but when the fly ash content continued to increase, the self-shrinking strain also began to increase, and the change process is shown in the Figure 20 below. However, British scholar Haque et al. [117] found that the ultimate shrinkage value of fly ash concrete was 540–720 × 10^−6^, lower than or slightly higher than the benchmark concrete. Lee et al. [118] believed that fly ash partially replaced cement and significantly reduced ECC self-shrinkage, but fly ash alone could not avoid early cracks. According to Malhotra et al. [119] research results, high-performance concrete with effective fly ash content has good volume stability and better resistance to temperature shrinkage, self-shrinkage, and ten-shrinkage cracking compared with Bochylan cement concrete. However, its plastic shrinkage cracking tendency is more evident under the lack of curing. Therefore, the content of fly ash in ECC should be controlled to achieve the best dry shrinkage.

## 6. Constitutive Structure of ECC

It is necessary to study the basic mechanical properties of ECC material level intensely if ECC material is used in building structures. Therefore, establishing the ECC constitutive model is crucial for studying ECC components and even the structural level. Therefore, the two typical ECC constitutive were introduced: tension constitutive and compression constitutive.

### 6.1. Uniaxial Compression Constitutive of ECC

Due to the brittleness of concrete material, it is mainly used to withstand pressure in practical engineering. According to the ECC dense accumulation principle, the usual coarse aggregate in traditional concrete is removed, which makes the matrix structure dense and has higher compressive performance and durability. Therefore, it is necessary to explore the law of ECC’s compressive performance [120]. The uniaxial compression test method is mature and simple to operate. It can measure the peak strength, peak compressive strain, and compressive strength and obtain the relationship between stress and strain. It is the most basic test method for measuring compressive strength. Scholars at home and abroad analyzed ECC’s mechanical properties, drew the stress-strain curves through uniaxial compression experiments, and established the uniaxial compression constitutive models with different characteristics.

**Figure 21:**
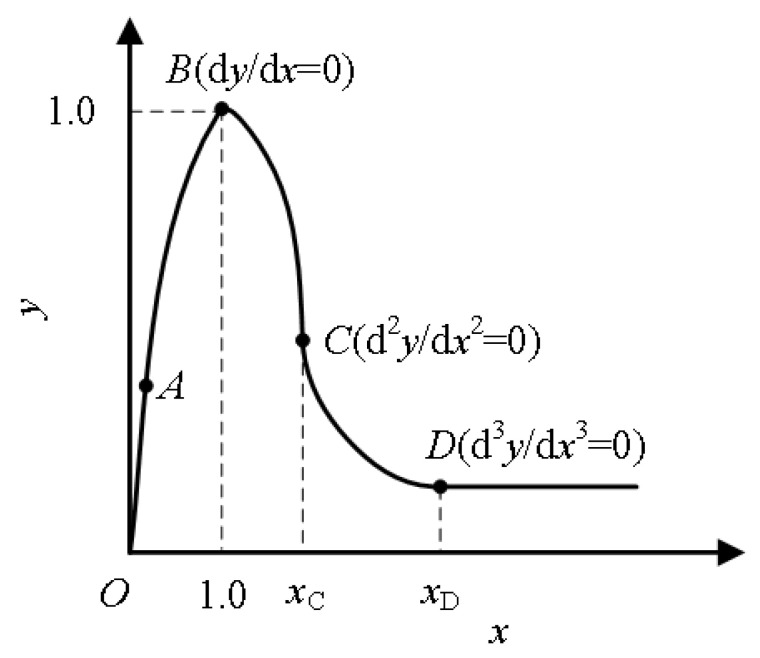
Uniaxial compression stress-strain full curve of ECC (no-dimensional) [121].

Through uniaxial compression tests, domestic and foreign researchers generally obtain the dimensionless stress-strain curve, as shown in the Figure 21 below. The first stage of OA is the linear elastic stage, where the stress and strain develop in proportion, and its slope is the material’s elastic modulus. The second stage AB is the yield stage, where the stress-strain curve is no longer linear, and the specimen begins to produce plastic deformation. When the stress reaches the maximum value, the corresponding stress is called the peak compressive strength or compressive strength, and the cracks in the ECC slowly expand at this stage. The third stage of BD is the ductility stage. The specimen begins to expand laterally at the same time as compression, and the crack expands rapidly and widens, finally forming a through-oblique crack. Finally, in the fourth stage, after point D, there is a residual stress stage. The crack width continues to increase, but due to the presence of fibers, ECC cracks without breaking and can still bear part of the load.

**Figure 22:**
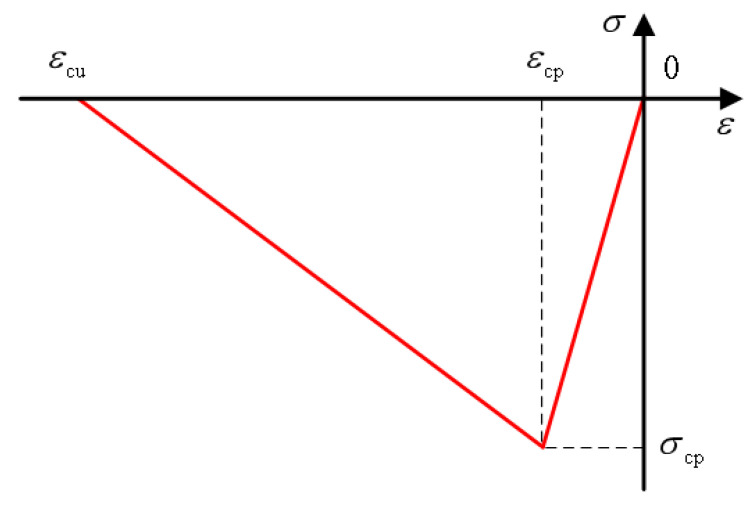
Han’s uniaxial compression constitutive model [124].

**Figure 23:**
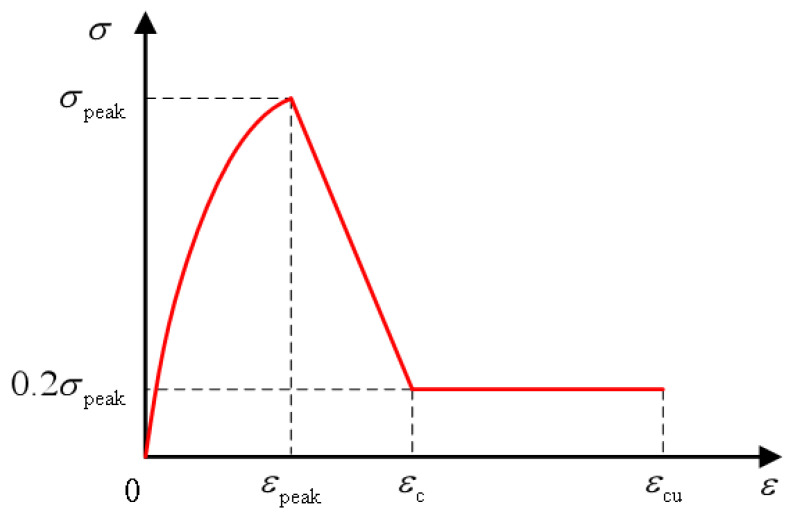
Xu ’s uniaxial compressive constitutive model [125].

Researchers at home and abroad have simplified stress-strain curves to varying degrees and established corresponding constitutive models, which can be divided into linear and nonlinear models. Zhu et al. [122] built a linear model with the ratio of axial compressive strength to 100 mm cube compressive strength *α* = *f_c_*/*f_cu_*. Qin [123] has the linear equation built up by Sargin and Saenz models. The Han double-line and Xu uniaxial compression models are the most representative linear models. Han [124] directly simplified the stress-strain curve into the double-broken line model, as shown in the Figure 22 below. Oblique straight lines expressed the strain, and the expression was shown in the formula. This constitutive model is simple in form and easy to calculate. However, because it does not consider the residual strength of ECC material, it is different from the stress-strain obtained in the experiment. The uniaxial compression constitutive model proposed by Xu et al. [125] team considers the residual strength of ECC materials, which adopts quadratic parabolic expression in the rising section and double-fold line expression in the descending section, as shown in the Figure 23 below. He defines the residual strength of ECC materials as 20% of the peak strength. See the diagram below for the specific diagram and the constitutive equation below. Compared with Han’s model, the uniaxial compression model of Xu has a more complex and complicated calculation but a higher degree of fitting. Therefore, readers can choose different constitutive models with the desired accuracy.

(1) $$\sigma =\{\begin{array}{c} E_{0}\varepsilon \;\;\;\;\;\;\;\;(0\leq \varepsilon <\varepsilon_{cp})\\ \sigma_{cp}-\sigma_{cp}\frac{\varepsilon -\varepsilon_{cp}}{\varepsilon_{cu}-\varepsilon_{cp}}\;\;(\varepsilon_{cp}\leq \varepsilon <\varepsilon_{cu})\\ 0\;\;\;\;\;\;\;\;\;\;\;\;\left(\varepsilon_{cu}\leq \varepsilon \right) \end{array}$$

$E_{0}$ is the initial modulus of ECC component; $\sigma_{cp}$, $\varepsilon_{cp}$ is the peak stress of ECC member and the corresponding peak strain.

(2) $$\sigma =\{\begin{array}{l} \sigma_{peak}\left[\frac{2\varepsilon }{\varepsilon_{peak}}-{\left(\frac{\varepsilon }{\varepsilon_{peak}}\right)}^{2}\right]\;\;\;\;\;\;\;\;\;\;\;\;\;\;\;\;\;\;\;\;\;\;\;\;0\leq \varepsilon <\varepsilon_{peak}\\ \frac{\sigma_{peak}}{\varepsilon_{c}-\varepsilon_{peak}}\left(\varepsilon_{c}-0.2\varepsilon_{peak}-0.8\varepsilon \right)\;\;\;\;\;\;\;\varepsilon_{peak}\leq \varepsilon <\varepsilon_{c}\\ \;\;\;\;\;\;\;\;\;\;\;\;\;\;\;\;\;0.2\sigma_{peak}\;\;\;\;\;\;\;\;\;\;\;\;\;\;\;\;\;\varepsilon_{c}\leq \varepsilon \end{array}$$

$\sigma_{peak}$, $\varepsilon_{peak}$ are the peak stress of the curve and the corresponding peak strain; $\varepsilon_{c}\;$is the strain where the stress decreases to 20% of the peak stress.

For the nonlinear analysis model, Guo [126] proposed using piecewise expression to describe the stress-strain curve characteristics of concrete and using polynomials to describe the ascending stage. The specific formula is as follows Equation 3. As a result, the ascending and descending changes are continuous, with an excellent fitting effect. Considering the mathematical description of the geometric features of the entire stress-strain curve under compression, Li et al. [127] took the undetermined parameter A as the ratio of the initial elastic modulus to the tangent modulus at the peak stress, the specific formula is as follows Equation 4 and the curve equations they proposed were shown in the formula respectively.

(3) $$y=\{\begin{array}{ll} \frac{\mathrm{Ax}-x^{2}}{1+\left(A-2\right)x} & 0\leq x<1\\ \frac{\mathrm{Ax}}{1+\left(A-2\right)x+x^{2}} & \;\;\;\;x\geq 1 \end{array}\;$$

(4) $$y=\{\begin{array}{cc} \alpha_{a}x+\left(3-2\alpha_{a}\right)x^{2}+\left(\alpha_{a}-2\right)x^{3} & \left(0\leq x\leq 1\right)\\ \frac{x}{\alpha_{d}{\left(x-1\right)}^{2}+x} & \left(x\geq 1\right) \end{array}$$

### 6.2. Uniaxial Tension Constitutive of ECC

**Figure 24:**
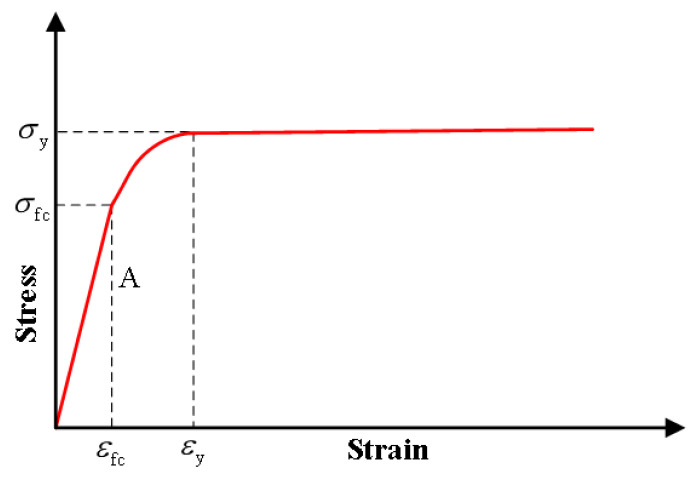
Typical ECC tensile stress and strain curve [51].

The tensile strength essentially controls the mechanical behavior of structural members. In practical engineering, the calculation of the bearing capacity of structural members, such as shear, torsion, and cracking, is related to the tensile strength of concrete [128]. The excellent tensile performance of ECC is an essential feature that distinguishes it from other high-performance concrete, so the tensile performance is significant. The principle of the axial tension test is simple, which can not only measure the tensile strength and ultimate tensile value but also obtain the relationship between stress and strain. So far, the uniaxial tension test is still the most basic test method [129]. The stress-strain curve generally obtained by domestic and foreign scholars through uniaxial tensile tests is similar to Li [121], as shown in Figure 24.

**Figure 25:**
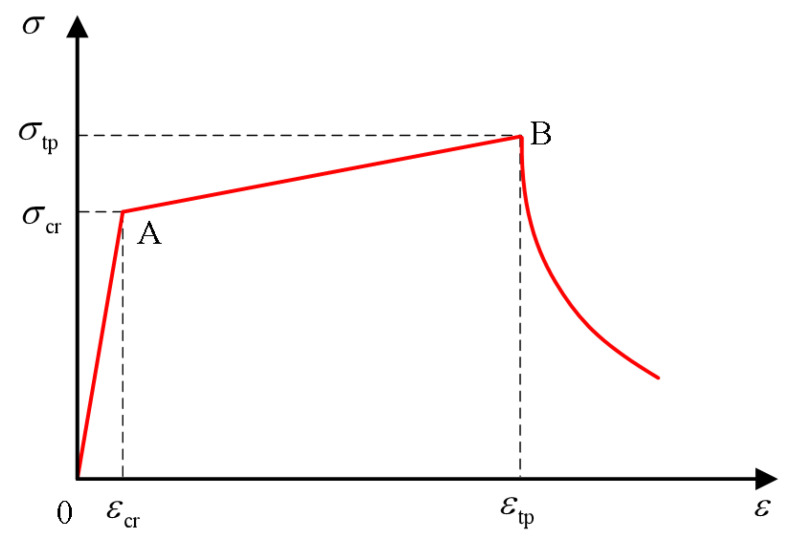
Kanda et al. ‘s uniaxial tensile constitutive model (ignoring strain softening) [130].

Scholars at home and abroad analyzed the mechanical properties of ECC, drew the stress-strain curves through uniaxial tensile experiments, and established various uniaxial compression constitutive models. The most representative is foreign scholar Kanda et al. [130], who proposed A double-broken line model of uniaxial tensile stress-strain relationship based on many ECC tensile test results. According to the first crack point A and peak point B on the curve, Kanda et al. divided the curve into three stages: linear elastic section, multi-crack development section, and strain softening section. The three stages are shown in Figure 25.

After ignoring the expression of the strain-softening segment, the constitutive equation for the first two stages is:

(5) $$\sigma =\{\begin{array}{c} E_{0}\varepsilon \;\left(\varepsilon \leq \varepsilon_{cr}\right)\\ \sigma_{cr}+\left(\sigma_{tp}-\sigma_{cr}\right)\frac{\varepsilon -\varepsilon_{cr}}{\varepsilon_{tp}-\varepsilon_{cr}}\;\;\left(\varepsilon_{cr}\leq \varepsilon \leq \varepsilon_{tp}\right) \end{array}$$

**Figure 26:**
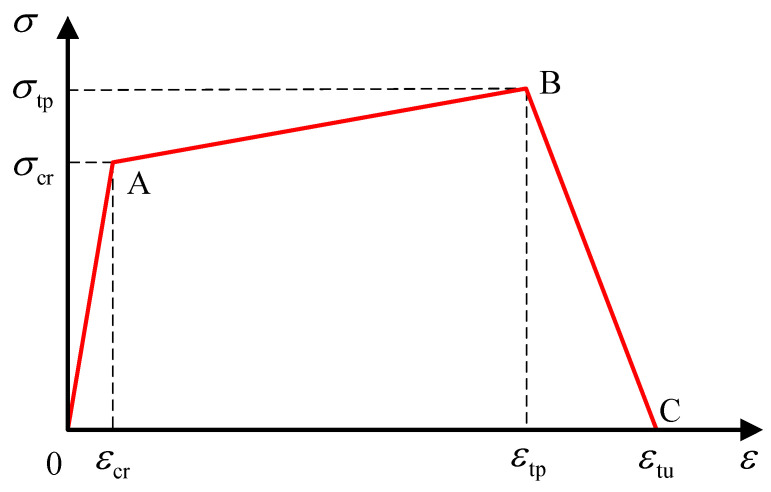
Uniaxial resistance accumulator model of Han (considering strain softening) [124].

$\sigma_{cr}$, $\varepsilon_{cr}$ are respectively the stress and strain of ECC material when the first crack occurs; $\sigma_{tp}$, $\varepsilon_{tp}$ are respectively the stress and strain c of ECC material at peak state; $E_{0}$ is the initial elastic modulus of ECC component at the elastic stage. Han et al. [9] took the expression of the first two sections of the curve to be the same as Kanda in ECC numerical simulation analysis as shown in the Figure 26, and expressed the curve of the strain-softening section between the peak point ($\varepsilon_{tp}$, $\sigma_{tp}$) and the ultimate tensile strain point ($\varepsilon_{tu}$, 0) as follow:

(6) $$\sigma =\{\begin{array}{c} \begin{array}{c} \;E_{0}\varepsilon \;\;\;\;\;\;\;\;\;\;\;\;\;\;\;\;\;\;\;\;\;\;\;\;\;\;\;\;\;\;\;\;\;\;\;\;\;\left(\varepsilon \leq \varepsilon_{cr}\right)\\ \sigma_{cr}+\left(\sigma_{tp}-\sigma_{cr}\right)\frac{\varepsilon -\varepsilon_{cr}}{\varepsilon_{tp}-\varepsilon_{cr}}\;\;\left(\varepsilon_{cr}\leq \varepsilon \leq \varepsilon_{tp}\right) \end{array}\\ \sigma_{tp}-\sigma_{tp}\frac{\varepsilon -\varepsilon_{tp}}{\varepsilon_{tu}-\varepsilon_{tp}}\;\;\;\;\;\;\;\;\;\left(\varepsilon_{tp}\leq \varepsilon \leq \varepsilon_{tu}\right) \end{array}$$

**Figure 27:**
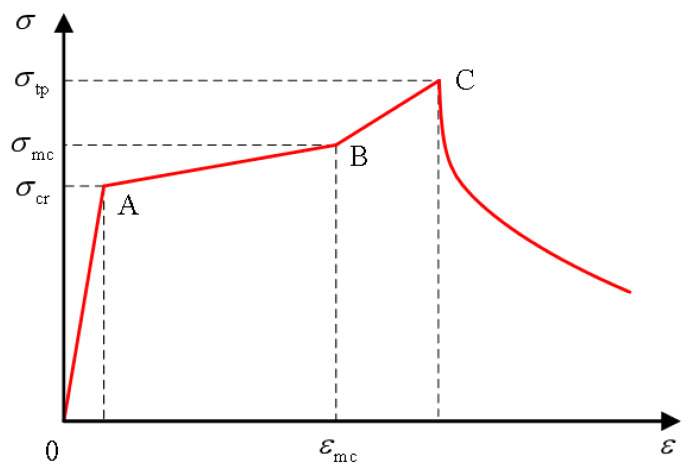
Xu et al. ’s uniaxial anti pull-constitutive model [131].

Xu et al. [131] proposed that the curve can be simplified into a three-line model, as shown in Figure 27.

When the specimen is at the starting point B of the rear multi-crack development section, the stress transmitted by the fiber at the crack interface cannot cause the matrix to generate new cracks. It is because the bearing capacity of the cement matrix has been completely lost. The stress is all borne by the fiber. With the increase of strain, the fibers at the crack interface are gradually pulled off or pulled out from the matrix. Due to the slip-hardening effect of the fibers, the stress of ECC material gradually increases until the material’s tensile strength is reached, the curve reaches peak point C, and the multi-crack opening section ends. Therefore, the expression of the three-fold stress-strain curve of Xu [131] is as follows:

(7) $$\sigma =\{\begin{array}{c} \begin{array}{c} E_{0}\varepsilon \;\left(\varepsilon \leq \varepsilon_{cr}\right)\\ \sigma_{cr}+\left(\sigma_{tp}-\sigma_{cr}\right)\frac{\varepsilon -\varepsilon_{cr}}{\varepsilon_{tp}-\varepsilon_{cr}}\;\;\left(\varepsilon_{cr}\leq \varepsilon \leq \varepsilon_{tp}\right) \end{array}\\ \sigma_{mc}+\left(\sigma_{tp}-\sigma_{mc}\frac{\varepsilon -\varepsilon_{mc}}{\varepsilon_{tp}-\varepsilon_{mc}}\right)\;\;\;\;\;(\varepsilon_{mc}<\varepsilon \leq \varepsilon_{tp}) \end{array}$$

$\sigma_{mc}$, $\varepsilon_{mc}$ are respectively the stress and strain of ECC at the end of multi-crack cracking. The remaining letters are the same as above. Furthermore, the above three-fold or two-fold models do not consider the expression of the strain-softening curve after the peak point.

The constitutive mechanical structures of engineering fiber cementitious composites have been summarized, and their characteristics, advantages, and disadvantages are included in Table 2.

## 7. Durability of ECC

Under normal conditions, the structure layout is reasonable if the concrete structure design is proper and the construction quality is reliable. As a result, the structure has good working performance. However, when the structure suffers from various external uncontrollable factors such as accidental load, structural function change, deterioration of service conditions, or improper maintenance, the applicability, durability, and safety of the concrete structure will be affected. Therefore, studying ECC’s durability for future ECC applications is essential.

### 7.1. Resistance to Freeze-Thaw Properties

One of the most dangerous environmental conditions for concrete is the freeze-thaw cycle. In the cold area of north China, the freeze-thaw cycle is often the main factor leading to the deterioration and destruction of building structures. Fatigue stress quickly occurs in the building structure damaged by the freeze-thaw cycle, which leads to internal damage and strength reduction. If it acts on the pavement, it will cause surface erosion and many cracks. In the bridge, it will cause cracking of the concrete surface layer, denudation of mortar, and exposure of coarse aggregate, as well as exposure and corrosion of reinforcement [132]. To solve the above problems, scholars studied the freeze-thawing resistance of ECC. Jang et al. [133] found that after 300 freeze-thawing cycles, the compressive strength of ECC was reduced by 25%. Compared with ordinary concrete and steel fiber concrete, ECC fracture modulus has little change during the freeze-thaw cycle. The study by Leah et al. [134] also showed that even after 300 freeze-thaw cycles, the tensile strain capacity of ECC samples was still more than 2%. Therefore, it is concluded that ECC has stronger freeze-thaw resistance than concrete, and the future application trend is to replace concrete to improve the freeze-thaw resistance.

### 7.2. Impermeability Performance

Cracks are inevitable in the concrete structure; water, corrosive substances, and oxygen will enter the concrete through cracks and corrosion, thus reducing the structure’s service life. Lepech et al. [135] studied the impermeability of ECC material to improve impermeability and avoid cracks. When the crack width decreased from 550 μm to less than 100 μm, the permeability coefficient of water decreased by seven orders of magnitude, which was much stronger than the impermeability of concrete. The research results of Li et al. [136] on the water permeability of ECC show that ECC still has good impermeability when the crack width is between 40 and 70 μm, and the permeability coefficient of ECC presents a gradually increasing trend with the increase of the crack width. The excellent impermeability of ECC can effectively prevent the penetration of harmful erosive media. The fibers in the fiber-reinforced cement base form a uniform directional support system in the ECC, which can effectively prevent micro-cracks from developing into fine cracks, thus improving impermeability [137]. The unique impermeability of ECC can better replace the role of concrete in impermeability. It can strengthen the subsequent research on constructing ECC materials in roads and bridges.

### 7.3. Fatigue Resistance Performance

ECC structure is subjected to two types of fatigue loads: one is dynamic effects such as vehicles, earthquakes, wind, and waves; the other is due to the dramatic changes in the external environment, which makes the structure experience temperature difference and freeze-thaw repeatedly, thus generating indirect cyclic stress inside [138]. Under repeated cyclic load, the structural resistance due to the damage and failure of building materials will decline with the accumulation of fatigue damage. It resulted in fatigue failure of the structure under static load strength and reduced reliability [139], which seriously endangers normal and safe building structure use. For roads, fatigue damage usually begins with surface cracking, and the fatigue load caused by vehicle driving often makes the cracking more serious. Leung et al. [140] conducted the anti-fatigue performance test of ECC. Replacing concrete in the tensile area with ECC can improve fatigue strength and prolong fatigue life. The thicker the ECC is, the more pronounced the enhancement effect will be. Lee et al. [141] believed that fiber was beneficial in improving the anti-fatigue performance of concrete. According to the latest research, due to its excellent mechanical properties, weather resistance, blast resistance, impact resistance, water resistance, and other physical and chemical properties, polyurea coating (PC) has been widely used in various fields of life and production of substrate waterproof anti-corrosion. PC exhibits excellent physical and mechanical properties and good adhesion to the substrate structure. Furthermore, the adequate vibration resistance of composite materials mixed with appropriate PC shows unique advantages in military protection and aerospace [142].

Chai et al. [143] conducted fatigue tests on ECC and found that cracks reached the most apparent size when the loading load peaked. However, no significant shedding area occurred, and only small debris fell, indicating that ECC had a good anti-fatigue performance. Therefore, ECC’s excellent anti-fatigue performance makes it a desirable material for fatigue structures.

## 8. Conclusions

In this paper, the development prospect of engineering fiber cementitious composites is first introduced. Then the basic mechanical properties and durability of engineering fiber cementitious composites are studied by uniaxial compression, uniaxial tensile, and conventional bending tests, respectively, according to fiber types, mineral admixture types, water-binder ratio, and other factors. According to the stress-strain curve, various constitutive equations suitable for PVA-ECC are proposed. Through the above research, the main conclusions are as follows:

1. Uniaxial compression characteristics of PVA-ECC
  (1) With the increase in fiber content, the compressive strength of the PVA-ECC cube and the axial compressive strength are both improved. When the fiber content is between 1.5% and 2.5%, the overall compressive strength of the cement-based material will be increased. However, the compressive strength will decrease significantly when the fiber content exceeds 2.5%.
  (2) The increased water-binder ratio decreases compressive strength gradually. As a result, the relative proportion of cement becomes smaller, and it cannot fill the pores left by sand and stone aggregate, resulting in more significant porosity and gradually reduced compressive strength.
  (3) Within a specific range, the replacement of cement by fly ash has a positive effect on the compressive strength of ECC, which can be improved by about 20–30%. However, if the content exceeds a specific limit value, the compressive strength of ECC will decrease rapidly as the replacement rate of fly ash continues to increase.
2. Uniaxial tensile characteristics of PVA-ECC
  (1) The cracking strength, cracking strain, ultimate tensile strength, and ultimate tensile strain of the ECC thin plate increase with the increase of PVA fiber volume content. For example, when PVA fiber volume content is 2.0%, the bridging effect of fiber can be fully exerted, and the tensile performance of ECC is also the most significant improvement.
  (2) With the increase of the water-binder ratio, the ultimate tensile strength of PVA-ECC decreases at 7 and 28 d, but the tensile strain hardening characteristics and ductility of PVA-ECC are obviously improved. Therefore, the water-binder ratio should be selected when preparing PVA-ECC.
  (3) When mineral admixture is added to ECC, the ultimate tensile strength decreases, but the ultimate tensile strain increases with the admixture amount.
3. Bending strength of PVA-ECC
  (1) With the increase of PVA fiber volume content (1.0%→2.0%), the toughness index of ECC firstly increases and then decreases and reaches the maximum at 1.5%. Moreover, increasing PVA fiber content can improve the initial crack strength and strain hardening effect when the first crack appears.
  (2) With the increase of fly ash content, the crack width and free drying shrinkage of the ECC structure decrease significantly. As a result, the interfacial bonding force between PVA fiber will be improved effectively.
4. Compared with conventional concrete, ECC has strong freezing resistance, permeability resistance, and anti-fatigue performance, which makes ECC have a broad application market and occupy a place in nowadays bridge construction, pavement laying, and other aspects.

## 9. Future Expectations

This paper summarizes the mechanical properties of ECC, summarizes the conclusions from three aspects: fiber type, water-cement ratio, and mineral admixture, respectively, in terms of compressive resistance, tensile resistance, and bending resistance, and classifies and summarizes the research results of domestic and foreign scholars. It is found that the current research on ECC needs to be improved in the following aspects, and further research can be conducted from these aspects. Furthermore, to better develop the scope of future ECC applications.

(1) At present, scholars at home and abroad focus on studying the mechanical properties and durability of PVA-ECC. However, there is still a need for more exploration of other fibers, such as basalt fiber and carbon fiber, there still needs to be more systematic and comprehensive research, and the research level only stays on the surface. Therefore, it is expected to find cheaper and better-performance fibers to replace PVA fiber in the market by increasing the research on other fibers.
(2) More experiments must be conducted on mixing hybrid fibers into a cement base. Currently, PVA-steel fibers are often mixed for experiments at home and abroad, and the data needs to be more comprehensive. The subsequent research on single fiber can be expanded to study hybrid fibers.
(3) The water-binder ratio of ECC is complex in mechanical research. Domestic and foreign scholars have reached conclusions by changing the water-binder ratio in mechanical tests, but opposite conclusions have occurred many times. Therefore, researchers should continue to study the theoretical and practical points of the influence of the water-binder ratio on mechanical properties in the future to form a set of confirmed water-binder ratio theories.
(4) To replace cement with mineral admixtures, fly ash, and silica fume have been fully studied. Subsequently, studies can be conducted on waste ceramic powder and glass powder to enhance the utilization of solid waste materials. Furthermore, reclaimed micro powder is an emerging research project nowadays. For example, the reclaimed brick powder studied in Tian [1] can not only significantly solve urban construction waste but also can be applied to the characteristic material arresting system of airport runway safety zone due to the low strength characteristics of the generated reclaimed micro powder ECC [144].

## Figures and Tables

**Table 1:** Physical indexes and advantages of different fibers.

Kind	Pattern	Density/(g/cm^3^)	Diameter/μm	Tensile Strength/MPa	Modulus of Elasticity/GPa	Limit Elongation Rate/%	Merit
PVA Fiber [34,35]	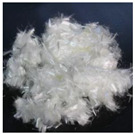	1.3	39	1600	39	7	High strength, high modulus, corrosion resistance, good dispersion
PP Fiber [36,37]	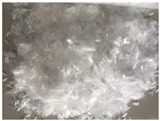	0.91	100	472	5.8	19.9	High strength, good ductility, good durability, and low price
CF Fiber [38,39]	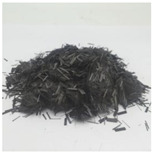	1.78	7	3530	230	1.5	The tensile strength and elastic modulus are very high and chemically stable
BF fiber [40]	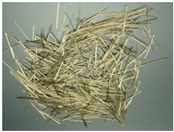	2.48	13	3800–4800	100	3.1	Higher tensile strength, deformation modulus, and corrosion resistance, can be directly degraded

**Table 2:** The mechanical constitutive structures of engineering fiber cementitious composites.

Classification	Classical Principal Structure Model	Feature
Uniaxially compressed	Han’s uniaxial compressive principal structure model	The form is simple and easy to calculate, but does not take into account the residual stress of the material.
	Xu’s Uniaxial compressive principal structure model	The form is complicated and the calculation is cumbersome, but the residual stress is taken into account and the fit is better.
Uniaxially tensioned	Kanda’s uniaxial tensile intrinsic structure model	Considering the crack development mechanism, the curve is divided into: linear elastic section, multi-slit development section, and strain softening section. The intrinsic constitutive equations of the first two stages are proposed, but the expression of the strain-softening section is neglected.
	Xu’s three-fold line principal structure model	The multi-seam developed segment of the presentational model proposed by Kanda was divided into the pre-multi-seam developed segment and post-multi-seam developed segment, and the post-multi-seam developed segment presentational equation was proposed.
	Han’s uniaxial tensile principal structure model	It considers the curve expression of the strain softening segment after the peak point.

## Data Availability

Not applicable.
